# Quantitative phase imaging based on holography: trends and new perspectives

**DOI:** 10.1038/s41377-024-01453-x

**Published:** 2024-06-27

**Authors:** Zhengzhong Huang, Liangcai Cao

**Affiliations:** Department of Precision Instrument, Tsinghua University, Beijing, 100084 China

**Keywords:** Imaging and sensing, Interference microscopy

## Abstract

In 1948, Dennis Gabor proposed the concept of holography, providing a pioneering solution to a quantitative description of the optical wavefront. After 75 years of development, holographic imaging has become a powerful tool for optical wavefront measurement and quantitative phase imaging. The emergence of this technology has given fresh energy to physics, biology, and materials science. Digital holography (DH) possesses the quantitative advantages of wide-field, non-contact, precise, and dynamic measurement capability for complex-waves. DH has unique capabilities for the propagation of optical fields by measuring light scattering with phase information. It offers quantitative visualization of the refractive index and thickness distribution of weak absorption samples, which plays a vital role in the pathophysiology of various diseases and the characterization of various materials. It provides a possibility to bridge the gap between the imaging and scattering disciplines. The propagation of wavefront is described by the complex amplitude. The complex-value in the complex-domain is reconstructed from the intensity-value measurement by camera in the real-domain. Here, we regard the process of holographic recording and reconstruction as a transformation between complex-domain and real-domain, and discuss the mathematics and physical principles of reconstruction. We review the DH in underlying principles, technical approaches, and the breadth of applications. We conclude with emerging challenges and opportunities based on combining holographic imaging with other methodologies that expand the scope and utility of holographic imaging even further. The multidisciplinary nature brings technology and application experts together in label-free cell biology, analytical chemistry, clinical sciences, wavefront sensing, and semiconductor production.

## Introduction

An accurate depiction of how waves spread in space and time is essential to the investigation of physical objects and their interactions with waves. In 1948, Dennis Gabor proposed the concept of holography^1^. After 75 years of development, holography has become a powerful tool for quantitative phase measurement. By using the reference wave, the sensor records the corresponding interference with the unknown object wave. The amplitude and phase of the object can be numerically reconstructed. Holographic recording and playback of waves have been used in numerous applications, including biological sample analysis, material representation, and material structure analysis^2,3^.

Phase refers to measuring the optical path length shift at each point in the field of view introduced by a specimen. The light absorption value of various semi-transparent objects is low because most of the light is scattering from the object, such as biological samples, resulting in a low-intensity-contrast image obtained by bright-field microscopy. The propagation of wavefront is described by the complex amplitude. Phase enables quantitative visualization of the inner structure or refractive index (RI) distribution of scattering samples with weak absorption. The main information can be reflected in the phase of the transmission wave due to the differences in the RI of cell liquid and external media. It is difficult for existing commercial cameras to achieve direct detection of phase in the visible light regime. To render the structures visible, one solution was to convert them into ‘amplitude objects’ using various stains or fluorescent tags with molecular specificity^4,5^. However, they are qualitative and sample-preparation-dependent, and the photobleaching and phototoxicity limit the fluorescent imaging of live cells. Furthermore, the use of exogenous labeling agents, such as fluorescent proteins or dyes, may alter the normal physiology of cells. The labeled cells cannot be re-injected into the human body. Another method is intrinsic contrast imaging occurred in the 1930s when Zernike invented a technique capable of imaging phase objects with high contrast and without the need for tagging^6^. The principle of Zernike’s phase contrast microscopy builds on Abbe’s understanding of imaging as an interference process^7^. To boost the contrast of the resulting interferogram and thus the image, Zernike added a phase shift of π/2. The additional phase shift places the scattered field in the antiphase with respect to the incident field. The resulting phase contrast field converts the phase into amplitude modulation. Phase contrast microscopes also balance the power of the two fields by attenuating the incident field. These simple modifications provides the microscope with the ability to visualize live, label-free cells and other weak absorption objects in rich detail.

Gabor showed that recording the intensity of the light emerging from an object at an out-of-focus plane incorporates both amplitude and phase information about the field at the image plane. After the publication of holography by Dennis Gabor in 1948 which led to receiving a Nobel Prize in 1971, several important holography-related inventions occurred in the 1960s. E. Leith’s work on off-axis holography^8^ had a substantial impact in making holography a much more practical and popular discipline. The invention of the laser made holography even more practical. A. Lohmann’s introduction of computer-generated holograms used computers to numerically generate holograms to be printed and photographed for optical reconstruction^2,9^. In the late 1960s, there was the invention of digital holography (DH) by J. W. Goodman, who proposed using electronic recording of holograms followed by numerical processing to reconstruct the object digitally^10^. When a recording film is illuminated by the same incident wave, the image of the object is recreated at a certain distance away from the film. The impact of DH on microscopy became significant much later in the 1990s when charge-coupled devices (CCD) were available as detectors. In 1994, Schnars and Jüptner demonstrated “lensless” off-axis DH using a CCD as a photoelectric detector^11^. Soon after, the benefits of DH were exploited in microscopy by several groups. It produces a new dimension of observation about cells, providing the ability for quantitative phase imaging (QPI). The recording media and image reconstructions are now digital, and the field is known as DH^12,13^. Its application to microscopy is known as digital holographic microscopy (DHM)^14^. Due to the quantitative reconstruction of the scattering field, DH has been endowed with multidisciplinary application from visible light to other bands^15,16^, enabling it to bring technology and application experts together in cell biology, analytical chemistry, clinical sciences, medical imaging, and tissue engineering.

With the developments of hardware and data processing ability, DH has produced various branches. In this review, we introduce the fundamental problem of DH and trace the development of methods to solve this problem. With the increasing availability of computational resources, these solutions have increasingly converged, leading to several key applications in quantitative biology and a dramatic increase in research interest in DH. We discuss the history, techniques, and advances in DH. It shapes future applications of this technology for addressing opportunities and challenges in production and biomedicine. We conclude by discussing the main ongoing DH research areas.

### The fundamental problem of digital holography

**Fig. 1:**
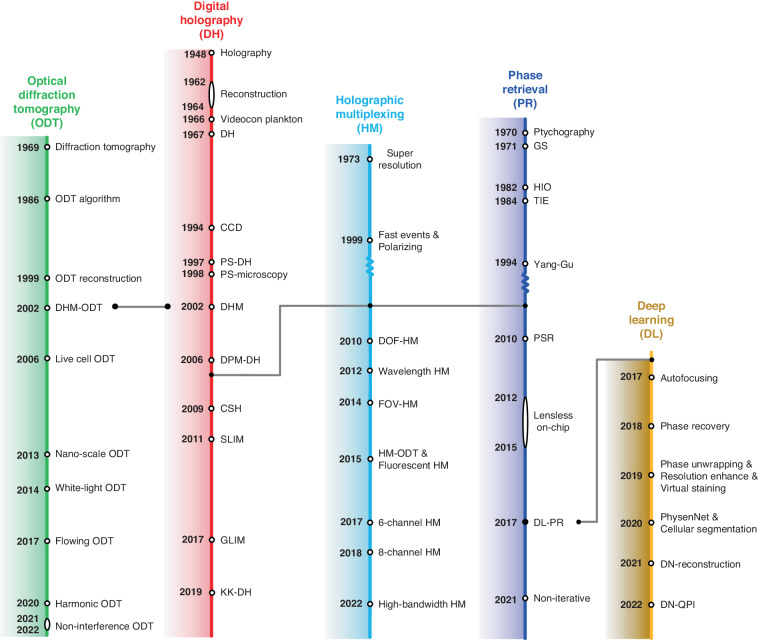
Holography has undergone a steady increase in interest driven by advances. Schematic of four main holographic approaches with digital holography, phase retrieval, holographic multiplexing, and optical diffraction tomography is indicated. These methods have improved extensively over time with the emergence of greater computational resources. The progress of the main events is shown in the timeline. The citations are shown in the following: Digital holography (DH): Holography^1^, Holographic reconstruction^8,35,36^, Videocon plankton^9,37^, Digital holography (DH)^10^, Charge-coupled device (CCD)^11^, Phase shifting digital holography (PS-DH)^49,63,67,68^, Phase shifting microscopy (PS-microscopy)^442^, Digital holographic microscopy (DHM)^443^, Diffraction phase microscopy (DPM-DH)^168^, Compressive sensing holography (CSH)^140^, Spatial light interference microscopy (SLIM)^80^, Gradient light interference microscopy (GLIM)^82^, Kramers–Kronig digital holography (KK-DH)^43^. Optical diffraction tomography (ODT): Diffraction tomography^18^, Tomography algorithm (ODT algorithm)^444^, Tomographic reconstruction (ODT reconstruction)^445^, Tomography based on DHM (DHM-ODT)^446^, Live cell tomography (Live cell ODT)^447^, Nano-scale tomography (Nano-scale ODT)^448^, White-light tomography (White-light ODT)^81^, Flowing tomography (Flowing ODT)^277^, Harmonic tomography (Harmonic ODT)^259^, Non-interference tomography (Non-interference ODT)^245^. Phase retrieval (PR): Ptychography^122^, Gerchberg-Saxton (GS) algorithm^40^, Hybrid input-output (HIO)^96^, transport of intensity equation (TIE)^449^, Yang–Gu algorithm^97^, Pixel super-resolution (PSR)^154^, Lensless on-chip imaging^87^, Deep-learning phase retrieval (DL-PR)^165,450^, Non-iterative reconstruction^45^. Holographic multiplexing (HM): Super-resolution^214^, Polarizing imaging^451^, Fast events^175^, Depth of field multiplexing (DOF-HM)^182^, Wavelength multiplexing (Wavelength HM)^184^, Field of view multiplexing (FOV-HM)^194^, Fluorescent multiplexing (Fluorescent HM)^205^, Multiplexing for tomography (HM-ODT)^211^, 6-channel multiplexing (6-channel HM)^452^, 8-channel multiplexing (8-channel HM)^453^, High bandwidth multiplexing (High-bandwidth HM)^230^. Deep learning (DL): Autofocusing^454^, Phase recovery^165^, Phase unwrapping^327^, Resolution enhance^455^, Virtual staining^456^, PhysenNet^316^, Cellular segmentation^457^, Diffractive networks reconstruction (DN-reconstruction)^458^, Diffractive networks QPI (DN-QPI)^459^

The propagation of wavefront is described by the complex amplitude. Only the intensity-value measurement in the real-domain can be recorded by the camera due to the high-frequency oscillation of visible light. DH seeks to reconstruct the phase shift of a wave that passes through a sample^17^. Conventional optical detectors only respond to the intensity or amplitude of the incident light. How to calculate the phase shift becomes the necessary link with the help of additional optical configurations and computational algorithms. This is the fundamental problem of DH, which has stimulated the development of multiple DH techniques, as shown in Fig. 1. We discuss the development of DH in the context of these solutions, focusing on the primary approaches that have had the significant impact on modern holographic methods and applications: digital holography, optical diffraction tomography, phase retrieval, holographic multiplexing and deep learning.

**Fig. 2:**
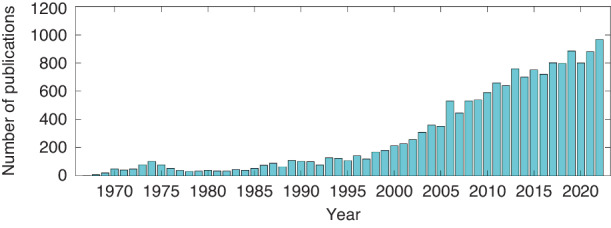
The growth in interest and advances in DH over time depicted by the number of publications, based on the Web of Science using search terms “Digital holography” by year. There was the invention of DH in 1967 by J. W. Goodman who proposed to use electronic recording of holograms followed by numerical processing to reconstruct the object digitally^10^. With the ever-increasing availability of computational resources, these significant technical solutions have increasingly converged. There was array-detector-based holographic imaging for applications until the 1990s

With the ever-increasing availability of computational resources, these solutions have increasingly converged, leading to several key applications in quantitative biology and a dramatic increase in research interest in DH, and the number of publications is shown in Fig. 2. The idea of digitally reconstructing the optical wavefront first appeared in the 1960s. The oldest study on the subject dates to 1967 with the article published by Goodman^10^. However, there was array-detector-based holographic imaging for applications until the 1990s^11^. In effect, there have been important developments in two sectors of technology: since this period, microtechnological processes have resulted in CCD arrays with sufficiently small pixels to fulfill the Shannon condition for the spatial sampling of a hologram; the computational treatment of images has become accessible largely due to the significant improvement in microprocessor performance, in particular their processing units as well as storage capacities. These developments are reflected in the increasing number of publications from the 1990s. Phase retrieval and holographic multiplexing are the advanced processes of computational reconstruction algorithm and optical configuration, respectively, which belong to the topic of DH. By combining the principle of computed tomography, the holographic reconstruction can be pushed to 3D imaging from multiple 2D measurements, achieving optical diffraction tomography (ODT), which was first theoretically proposed in 1969 by E. Wolf^18^ and followed by geometrical interpretation by Dändliker & Weiss^19^. The process of ODT is consistent with DH. Thanks to the advancements of laser sources, detecting devices, and computing powers, there have been experimentally significant technical advances in ODT. The applications have been expanded to various fields, from biophysics, cell biology, hematology, to infectious diseases^20^.

**Fig. 3:**
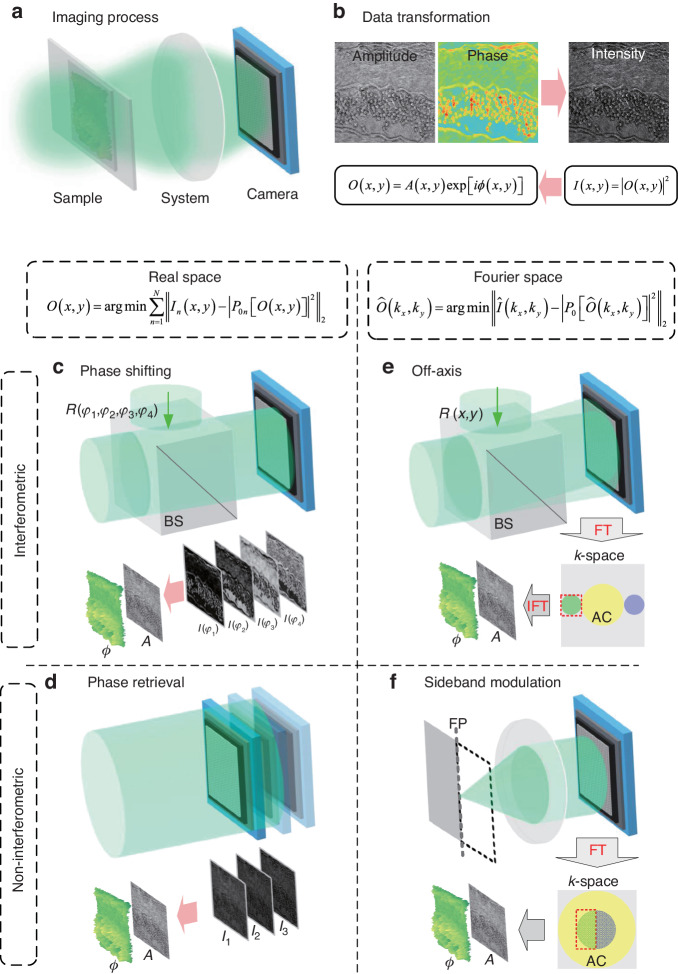
Fundamental methods of holographic imaging. Digital holographic recording and reconstruction are the processes of matrix conversion between the real-domain and the complex-domain. The spatial field distribution is complex-valued and the detections from the camera are real-valued. According to the configurations, the reconstruction methods can be classified as interferometric and non-interferometric methods. **a** Illustration of the imaging process. **b** Data transformation between the complex-amplitude wavefront and the intensity detection. **c** Phase shifting: In real-domain, using multiple intensities with different phase shifting to reconstruct amplitude and phase. BS: Beam splitter. **d** Phase retrieval: In real-domain, using multiple intensity images with different modulation to reconstruct amplitude and phase. **e** Off-axis holography: In the Fourier domain, using spatial carried frequency to modulate the band-limited wave in intensity image to reconstruct amplitude and phase. FT Fourier transform, IFT Inverse Fourier transform. **f** Sideband modulation: In the Fourier domain, using analytical sideband complex-amplitude wave to reconstruct amplitude and phase. FP Fourier plane

The “phase” of a periodic signal is a real-valued scalar in physics and mathematics, which describes the relative location within the span of each full period. The phase is typically expressed as an angle in degrees or radians. It varies by one full turn as the variable goes through each period in the monochromatic coherent optical field. In comparison to phase, the amplitude of the optical field is generally much easier to understand or comprehend. The square of the amplitude, also known as the “intensity” of light, is the only visible component with human eyes and imaging sensors, representing the energy of the light. The propagation of the optical wavefront is described by the complex amplitude. The complex-value in the complex-domain needs to be reconstructed from the intensity value measurement by the camera in the real-domain. A traditional imaging system is shown in Fig. 3a. The light illuminates the sample and then passes through an imaging system, and the sensor captures the images of the sample. One important reason is that the oscillation frequencies of light waves are around 10^14^ Hz, which is much higher than the response of human eyes (~30 Hz) or current imaging sensors (<10^8^ Hz). The phase is particularly prominent in some specific fields, such as optical metrology, material physics, adaptive optics, X-ray diffraction optics, biomedical imaging, etc. Most samples belong to phase objects with weak absorption in intensity. However, the spatial distribution of their RI or thickness is nonuniform with obvious features in phase. The acquisition of phase information is of particular importance, especially for label-free biological samples. The intrinsic contrast generated in phase is due to light scattering, which is the general term that describes the interaction between a field and the real part of the dielectric permittivity^21^. The phase expression can be derived from the far-zone scattered field using the wave equation, where the phase is linearly related to the object thickness and RI contrast in weak scattering approximation^22,23^. For the complex-amplitude wave, only the intensity can be recorded by the sensor, and the number of pixels in corresponding amplitude $A$ and phase $\phi$ become double, as shown in Fig. 3b. It becomes an ill-posed problem of the reconstruction from intensity to the complex-amplitude. In physics, the conservation of energy indicates that the reconstructed number of pixels needs to be smaller than the detected number of pixels. Two routes can be considered to transform the ill-posed to a well-posed problem. One route is in real-domain where multiple detections (*N* number) are required to confirm that the detected number of pixels is more than the number of pixels in amplitude and phase when *N* > 2. The other is in the Fourier domain where the band-limited condition of the wave is required to confirm that the detected area in the Fourier domain is more than that of amplitude and phase. ${P}_{0}$ is the projection operator between the object function and the diffraction wave in hologram. The bandwidth of object Fourier space is determined by the coherent transfer function (CTF) of the system, which is defined by the numerical aperture (*NA*) and the wavelength *λ*. For the real-domain, the first method is phase shifting based on interferometry. The holograms under different phase shifts in the reference path are recorded by the sensors. The amplitude and phase are reconstructed by using multiple holograms with phase shifting, as shown in Fig. 3c. The second method is phase retrieval based on non-interferometric detection. The measurements under different optical system parameters are used to reconstruct the amplitude and phase by using numerical algorithms, as shown in Fig. 3d. For the Fourier domain, the first method is off-axis holography based on interferometry. The complex-amplitude with a quarter of the pixel’s bandwidth can be separated with other unwanted terms like autocorrelation (AC) and conjugate spatial Fourier spectra, and then the object’s Fourier space can be filtered and reconstructed, as shown in Fig. 3e. The second method is sideband modulation based on non-interferometric detection. The complex-amplitude with a sideband modulation can be separated with its conjugate spatial Fourier spectrum, and then the amplitude and phase can be reconstructed by using the analyticity of sideband wave, as shown in Fig. 3f. The details of those principles and methods are introduced in the following.

### Overview of holographic reconstruction

**Fig. 4:**
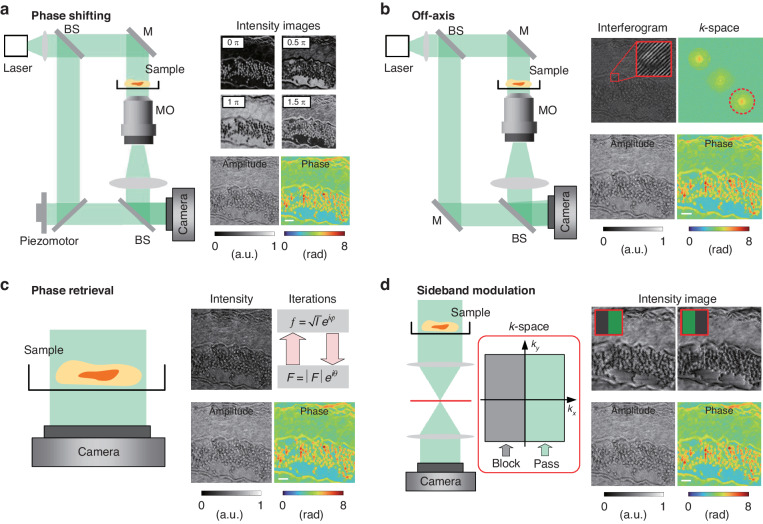
Examples of the four primary holographic imaging model. Those configurations are corresponding to the fundamental methods in Fig. 3. Only principles are shown here, and various variable experimental devices are produced based on them. **a** Phase-shifting holography: MZ interferometric example uses the interference between light beams passing through a sample and a reference to generate an interferogram. The reference field is in-line with the imaging field, and four phase shifting are considered phase reconstruction. MO: microscopic objective. **b** Off-axis holography: MZ interferometric example records the interference between an object wave and a reference wave, and the reference field is tilted to create spatial modulation. **c** Phase retrieval: non-interferometric imaging method that computationally reconstruct the phase shift from a sequence of intensity images taken under varying conditions. **d** Sideband modulation holography: A band-limited object wave with an upper half *k*-space is recorded by the camera with intensity-only, and the phase image is directly retrieved from an intensity image through the analytic expression

In DH, the most general method to calculate the phase is interferometry^24^. The incident wave is split into two paths. One path is considered as illumination on the sample to form a sample path, while another path is considered as a reference path, as shown in Fig. 4a, b, respectively. The interference relates to the phase shift of light passing through the sample with respect to the reference path. Interferometry was invented by Albert Michelson and it was further improved in collaboration with Edward Morley and famously used as the Michelson−Morley experiment^25^. The early improvements were the separation of sample and reference wave in the Mach−Zehnder (MZ) interferometer^26^ and the use of thin calcite films faced at 45° to enable micro interferometry^27^.

The next major advance in phase reconstruction toward biomedical applications was the calibration of a specific refractive increment using varying specimen compositions^28–31^, which enabled the calculation of the dry mass of label-free cells. A series of targeted improvement performances in biological applications have been reported. Interferometry provides reliable and reproducible quantitative data for internally complex mammalian cells. Although the relationship between amplitude and phase in an interferogram is straightforward, the requirements of a reference path increases the complexity and number of optical elements. The susceptibility to vibrations^32^ and instability of a light source are also increased^33^. Advances in several areas of interferometry-based measurements benefit from the increasing use of computers. Using phase shifting of reference path, the complex-amplitude can be reconstructed from multiple phase-shifting holograms, as shown in Fig. 4a. Digital emerged from the establishment of holography by Gabor for which he won the Nobel prize in 1971^34^. Gabor’s work demonstrated that light from a point source interfering with secondary waves from light scattered by an object produces a negative photograph of a 3D image. However, a conjugate image is also superimposed on the reconstructed image, resulting in ambiguity. The use of an off-axis reference wave can separate the real and conjugate images^8,35,36^. The configuration of off-axis holography is shown in Fig. 4b. The angle between the object and reference wave becomes larger than that in the conventional phase-shifting method. The irradiance of the interferogram is:

1 $$I(x,y)={I}_{R}+{I}_{O}(x,y)+2\sqrt{{I}_{R}{I}_{O}(x,y)}\cos [\omega x+\phi (x,y)]$$

where ${I}_{R}$ and ${I}_{O}$ are the intensity for the reference wave and object wave, respectively. *a* is the spatial frequency introduced by the off-axis angle *θ*, $\omega =2\pi \,\sin (\theta )/\lambda$, and *λ* is the wavelength. Because of the modulation frequency *ω*, the cross-term containing the phase can be isolated via FT, followed by a single sideband frequency filter. By filtering the sample term in the red circle and calculating with IFT, the complex-amplitude can be reconstructed from a single hologram. The DH provided an early application for living cells which were imaged using holography in a chamber^37^. The optical path for the sample can be used for a conventional microscopy. The diffraction limit of reconstruction is limited by the *NA* of the microscopic objective (MO). The advantage of off-axis methods is the single-shot capability, which allows high-speed imaging. But this boost in the time–bandwidth product comes at the expense of the space–bandwidth product (SBP). Both approaches are currently used broadly. The optimal choice depends on the application of interest.

Phase retrieval refers broadly to non-interferometric methods which computationally reconstruct the phase from a sequence of intensity images under varying conditions. As shown in Fig. 4c, the intensity of the object wave is directly projected on the camera under coherent or partially coherent illumination. They can be implemented using simple optical systems and enhance the performance of complex optical systems. It can be classified as either optimized or deterministic^38^. Iterative methods use iterative computation to enforce constraints in object between intensity images at the detector plane to address the phase problem^39^. The iterative methods were originally developed for electron microscopy to reconstruct the wavefront propagation between object and diffraction planes from the corresponding intensity images^40^. It iteratively approximates both source and target distributions from measured intensity images. However, it typically requires several iterations and is often stuck at local minima which is not the best solution. This limitation was partly addressed by the introduction of the steepest gradient search^41^ and input-output methods^42^.

By modulating the *k*-space, deterministic methods directly solve for holographic imaging without iterations and interferometric configuration, as shown in Fig. 4d. Sideband modulation holography describes a phase image in terms of an intensity image by utilizing the Kramers–Kronig (KK) relations in a band-limited imaging system^43,44^. By blocking half of the *k*-space, a band-limited object wave with an upper half *k*-space is recorded with intensity. The asymmetric *k*-space indicates the analyticity of the object wave in the upper half-plane. A phase image is directly retrieved from an intensity image through the analytic expression. By synthetizing the *k*-space from blocking one, the complex-amplitude with isotropic resolution can be reconstructed. The band-limited object wave can also be produced by changing the angle of illumination and placing unscattered wave at the edge of the pupil^43^. In this case, the two-dimensional (2D) synthetic-aperture phase imaging for resolution enhancement and 3D tomographic reconstruction are determinately endowed with non-interferometric configuration^45^.

## Modern phase measurement methods

### Phase shifting holography

**Fig. 5:**
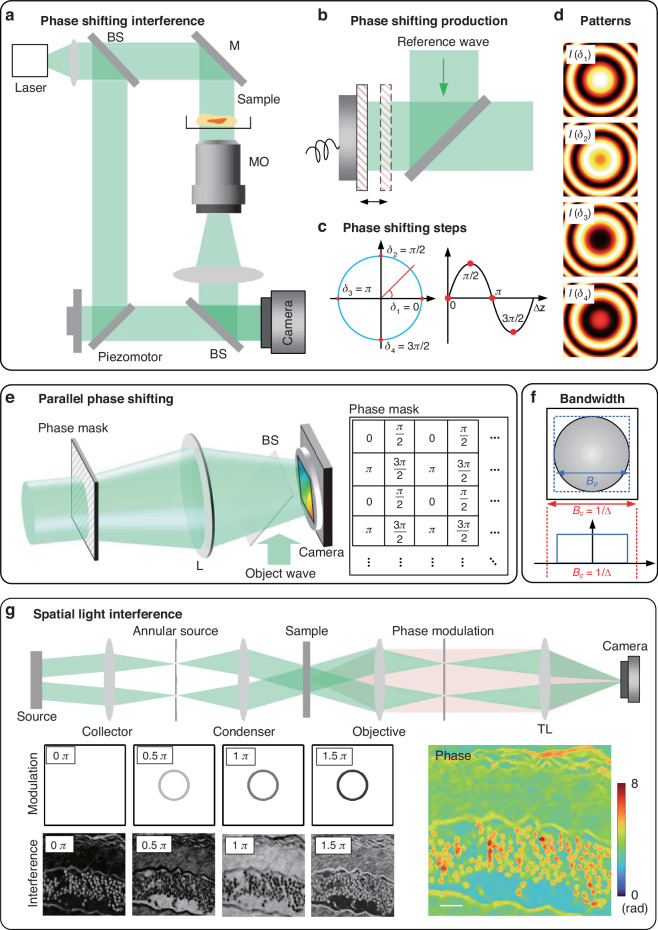
Phase-shifting holographic imaging model. The interferometry offers temporal phase shifts between the object field and the reference field. The spatial field distribution in the complex-domain is reconstructed from multiple interferograms in real-domain. It belongs to the real-domain interferometric method in Fig. 3c. **a** MZ interferometric configuration uses the interference between the object wave and reference wave. The reference field is in-line with the imaging field. **b** Phase-shifting production in the reference wave. **c** Phase shifting on the trigonometric circle and a sinusoidal function. **d** The phase shifting pattern of spherical phase example, where $\delta =0$, $\delta =\pi /2$, $\delta =\pi$, $\delta =3\pi /2$ are the steps of phase shifting, L: Lens. **e** The principle of parallel phase shifting technique. **f** The bandwidth limitation of phase-shifting holography. **g** Non-interferometric spatial light phase shifting technique. TL tube lens

The interferometry offers temporal phase shifts between the object wave and the reference wave. The spatial complex-amplitude field in the complex-domain is reconstructed from multiple interferograms with different phase shifts in real-domain. It belongs to the real-domain interferometric method in Fig. 3c, which preserves the SBP, at the expense of the time-bandwidth product^46^. The concept of phase shift was originally derived from the electronic engineering field in the 1960s, which was used to determine the phase difference between the two electrical signals^47^. In 1974, Bruning, J. H. et al. introduced this method into optical measurement^48^. Multiple interference images are acquired with varying subwavelength shifts in the reference relative to the sample optical path length^33^. In 1997, Yamaguchi I et al. introduced the phase shifting technology to DH^49^. A typical experiment setup is shown in Fig. 5a. Phase shifts are performed in the reference path using the piezo electric transducer in the initial model^49^. The piezo electric ceramic material produces a small variable under the external voltage driver. It pushes the plane reflector to generate corresponding displacement along the optical axis of the reference wave, as shown in Fig. 5b. The modulation elements can be achieved by digital devices such as a digital micromirror device (DMD) or a spatial light modulator (SLM)^50^, and analog methods such as polarization phase shifting^51^, diffraction phase shifting^52^, acousto-optic modulation phase shifting^53^, wavelength phase shifting^54^, tilt phase shifting^55^, etc. The larger phase shifts can be accurately measured by digitally combining images taken at two or more wavelengths^56^. Errors introduced from the unevenness of illumination wavefront need to be digitally corrected^57^. The irradiance at the detector is:

2 $${I}_{{\delta }_{n}}(x,y)={I}_{R}+{I}_{O}(x,y)+2\sqrt{{I}_{R}{I}_{O}(x,y)}\cos [{\delta }_{n}+\phi (x,y)]$$

where ${I}_{R}$ and ${I}_{O}$ are the irradiances for the reference wave and object wave, respectively, ${\delta }_{n}$ is the phase delay between the object arm and reference arm. The MZ based interferometry enables phase shifting recording by introducing the phase shift in the reference path. In general, the phase image *ϕ* can be determined from four intensity measurements corresponding to ${\delta }_{n}=0,\pi /2,\pi ,3\pi /2$ as

3 $$\phi (x,y)={\text{arg}}({I}_{0}-{I}_{\pi },{I}_{3\pi /2}-{I}_{\pi /2})$$

where $\text{arg}(x,y)$ is the counterclockwise angle from the positive *x* axis to the line that connects the origin and the point $(x,y)$^58^, as shown in Fig. 5c, d. Function $\text{arg}(x,y)$ is defined as

4 $${\text{arg}}(x,y)=\left\{\begin{array}{ll}{\arctan} (y/x) & x \,>\, 0\\ \pi /2 & x=0,y \,>\, 0\\ -\pi /2 & x=0,y \,< \,0\\ \pi +\arctan (y/x) & x\, <\, 0,y\,\ge\, 0\\ -\pi +\arctan (y/x) & x\, <\, 0,y\, <\, 0\end{array}\right.$$

Other numbers of frames have been used in phase-shifting interferometry^59^. Synchronous phase shifting technology can obtain multiple interferograms of phase shifting simultaneously. A direct method is that the different phase-shifting interferograms are recorded by multiple cameras^60–62^. This method can retain the high spatial resolution of reconstruction with no loss in temporal resolution. But a complicated optical configuration is required and it is sensitive to environmental interference. Another method is to divide different pixels for different phase shifting in a single camera, which is named simultaneous phase shifting (SPS). A specially-made phase mask covers the recording area of the camera^63–65^ so that each pixel corresponds to different phase steps, as shown in Fig. 5e. Four small adjacent squares on the mask board can be regarded as a phase shifting unit. The pixel units with the same phase steps are extracted to stitch into a new image by linear interpolation or other methods. Four interference patterns with different phase displacements can be obtained from a collection from the camera^66^. Other numbers of shifting steps have been used in SPS interferometry^63,67,68^. The final pixel resolution is only a quarter of the sensor^69^. The FOV segmentation can also be applied to obtain different phase shifts in addition to pixel mask segmentation. The segmentation modulation can be achieved by optical splitter devices such as prism^70–72^, grating^73–77^, Wollaston prism^78^, and Michelson interference structure^79^. The complex-amplitude with full pixel’s bandwidth can be reconstructed in the phase shifting method. As shown in Fig. 5f, the maximum allowable bandwidth can reach the sampling limit of the pixel. On-axis interferometry preserves the SBP at the expense of the time-bandwidth product^46^. But for the SPS method based on the pixel mask and FOV segmentation, it preserves the time-bandwidth product at the expense of the imaging sensor’s SBP.

The optical field at each point can be described as the interference between the scattered field and the incident field, which acts as a common reference for a highly parallel interferometry system. It inspires to achieve phase reconstruction from on non-interferometric configuration based on the phase shifting. In the 1930s, Zernike solved this problem by inserting a π/2 phase retarder in the objective pupil plane, introducing an extra π/2 phase delay between the incident and scattered fields^7^. Spatial light interference microscopy (SLIM) combines the spatial uniformity associated with white-light illumination and the stability of common-path interferometry. A schematic of the instrument setup is depicted in Fig. 5g. The SLIM was developed by producing additional spatial modulation to the image field outputted by a commercial phase contrast microscope^80^. The SLM in the add-on module provides further phase shifts in the pupil plane with increments of π/2. The active pattern on the SLM is calculated to precisely match the size and position of the objective phase-ring image. The phase delay between the scattered and unscattered waves is controlled and the four images corresponding to each phase shift are recorded by the camera. The phase in SLIM can be understood as that of an effective monochromatic field oscillating at the average frequency of the broadband fields^22^. The scattering potential is solved by deconvolving with the impulse response for white light under Born approximation. The axial distributions of the object are reconstructed by scanning the focus through the object, which enables the diffraction tomographic reconstruction under broadband illumination^81^. For the thick specimens, multiple scattering limits the contrast in optical 3D imaging. Gradient light interference microscopy (GLIM) based on phase shifting exploits a special case of low-coherence interferometry to extract phase from the specimen, which in turn can be used to measure cell mass, volume, surface area, and their evolutions in real time^82^. GLIM has all the benefits of common-path and white-light methods including nanometer path-length stability, speckle-free, and diffraction-limited resolution. Based on the Wolf equations for propagating correlations of partially coherent light, Wolf phase tomography based on phase shifting decouples the RI distribution from the thickness of the sample directly in the space-time domain^83^, without the need for FT and time-consuming deconvolution operations.

### Inline holography and phase retrieval

**Fig. 6:**
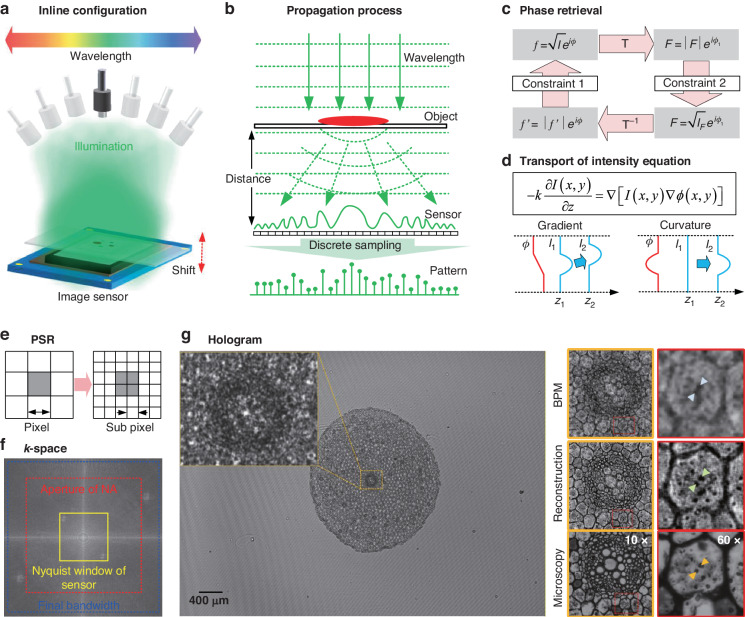
Lensless on-chip Inline (Gabor) holographic imaging model. The spatial modulations offer measurement diversity of detections. The spatial field distribution in the complex-domain is reconstructed from multiple detections in the real-domain. It belongs to the real-domain non-interferometric method in Fig. 3d. **a** Schematics of a lens-less on-chip Inline (Gabor) holographic microscope. A sequence of intensity images taken under varying conditions: different wavelengths, different recording distances, and different angles of illumination. **b** Configuration and propagation process of the in-line holographic imaging system. **c** Iterative phase retrieval for complex-amplitude from intensity images, T: transformation. **d** Phase retrieval for complex-amplitude reconstruction by TIE. The phase can be converted into intensity during diffraction, producing the transport of intensity effect. Phase slope induces the intensity translation, while phase curvature induces intensity convergence or divergence. **e** Pixel super-resolution (PSR) for the in-line on-chip holographic imaging. **f** The imaging system passbands in the Fourier domain. **g** Full FOV hologram of the specimen. For comparison, the typical imaging from conventional 10× and 60× MOs are shown^157^. BPM Back-propagation method

For centuries, biomedical imaging at the micron-scale has been powered by optical compound microscopes, leading to numerous discoveries at the micro- and nano-scale^84,85^. These conventional microscopes are operated with expensive and bulky lenses and other optomechanical parts, including alignment mechanics, and there is an inherent trade-off between the resolution and FOV. Over the last few decades, with the exponentially faster, cheaper, more powerful, and portable computational resources^86^, unconventional microscopy methods have emerged with simple and inexpensive hardware while relying on computation to digitally generate high-resolution images. Inline (Gabor) holography without introducing reference wave provides an effective path to calculate the weak absorption object directly from the wave propagation. Lensless on-chip microscopy^87,88^ has been extensively explored to bypass various limitations of a conventional compound microscope, providing much more compact, cost-effective, and wider FOV imagers. Instead of capturing a microscopic image of the sample directly, the image sensor records an in-line hologram under coherent or partially coherent illumination^2^, as shown in Fig. 6a. The original object, both its amplitude and phase images, are reconstructed digitally in inline holography^2,87^. The spatial complex-amplitude in the complex-domain is reconstructed from detections in the real-domain. It belongs to the real-domain non-interferometric method in Fig. 3d.

A weak absorption sample is placed on top of an image sensor with a typical distance of less than 1 mm, as shown in Fig. 6b. A partially coherent light source is used as illumination, usually at the distance of >2–3 cm above the sample. The light source can be a monochromator^89,90^, a laser^91,92^ in a benchtop system, or a light emitting diode (LED) in a portable device^88,93^, with an optional spectral filter^94^. The benefits of a partially coherent source include speckle reduction and multiple reflection interference noise, as well as much easier automated alignment of digital images^87,95^. In in-line (Gabor) holography, the object is assumed as weak absorption and it can be approximated as

5 $$t(x,y)=1+\Delta t(x,y)$$

where $\Delta t(x,y)\ll 1$. $t(x,y)$ is the transmittance function of the object. The object is locally illuminated by a plane wave *A*, and the exit wave propagates coherently over a distance *z*, which can be expressed as

6 $${P}_{z}[At(x,y)]={P}_{z}(A)+{P}_{z}[A\Delta t(x,y)]={A}_{0}+a(x,y)$$

where ${P}_{z}(.)$ is the free-space propagation operator over a depth of *z*. A hologram is formed by the interference of the scattered beam $a(x,y)$ with the unscattered wave ${A}_{0}$, and the intensity of this interference is recorded by the sensor:

7 $$I(x,y)={|{A}_{0}+a(x,y)|}^{2}={|{A}_{0}|}^{2}+{|a(x,y)|}^{2}+{{A}_{0}}^{\ast }a(x,y)+{A}_{0}{a}^{\ast }(x,y)$$

The FOV is approximately the entire active area of the image sensor under a unit magnification. The first term can be subtracted out using a background image. The second term can be ignored due to $\Delta t(x,y)\ll 1$. By performing back-propagation of Eq. (7), a focused image and a defocused image of the specimen can be obtained simultaneously. Phase retrieval methods solve the inverse problem from intensity measurements to the complex-amplitude of the sample, as shown in Fig. 6c. GS algorithm^40^ is the first practical iterative algorithm with two intensity images measured by replacing the computed amplitude distribution with prior measured amplitude. Although GS algorithm is widely used to reconstruct the phase on the object plane, the convergence speed decreases as the algorithm proceeds. To accelerate the convergence speed of the GS algorithm, hybrid input-output (HIO) algorithm has been proposed^96^. The phase retrieval issues can be treated as the model of high-dimensional ill-conditioned equations in the complex-domain^40,96–100^. Multiple-image phase retrieval added intensity data from the measurements by using different optical parameters. The intensities can be recorded at different heights^101,102^, wavelengths^103,104^, illumination angles^91^, illumination patterns^105^, etc. The general framework of this type of algorithm is called the GS iterative error-reduction method^40,96^. It is also referred to as the iterative projection method^106,107^, or the multi-height phase retrieval algorithm^92,101,108^. The multi-height phase retrieval process typically requires 6–8 heights to efficiently suppress the twin-image noise and other spatial artifacts. Because the phase retrieval problem is in general nonconvex, the algorithm may stagnate at a local optimum, which is also known as the phase stagnation problem^92,109^.

By using intensity image to reconstruct phase, the TIE is proposed which establishes the quantitative relationship between the longitudinal intensity variation and phase of a coherent beam with a second-order elliptic partial differential equation, as shown in Fig. 6d. Under the paraxial approximation and the weak defocusing approximation, the linearization of the relationship between the intensity and phase information can be realized^38^. Inline (Gabor) holography just records the diffraction intensities of the object wave with non-interferometric configuration. The phase is converted into intensity during diffraction, and this kind of intensity is often referred to as “phase contrast”, producing transport of intensity effect along the direction of propagation. Axial variation of intensity is determined by both phase slope and phase curvature, as shown in Fig. 6d. Phase slope induces the intensity translation, just like a prism, while phase curvature induces intensity convergence or divergence, just like a lens. The TIE is an effective tool to reconstruct phase distribution in inline (Gabor) holography. By using the determined boundary conditions, the phase can be reconstructed from multiple captured images at different axial distances. In the lensless system, the TIE is solved under simplified homogeneous when the FOV is large and most of the sample is distributed within the FOV, Neumann boundary conditions can be considered which assumes zero phase change at the image boundary^110,111^. However, it sometimes fails to provide an accurate solution that coincides with the exact phase^112–115^. The phase errors originate from the nonlinear components related to the finite difference approximation^112,113^, and the phase discrepancy associated with the TIE solvers. To compensate these inaccuracies, the reconstructed phase from TIE is used as an initial input for iterative optimization based on the GS-type algorithms^40,110^. Using this framework, lensless on-chip imaging of pathology slides with a image quality comparable to a high-end conventional compound microscope has been demonstrated^92,116^.

Multiple illumination angles can also be used for phase retrieval, meanwhile increasing the effective *NA* of reconstruction through the synthetic-aperture approach described earlier^91,117^. One common implementation of iterative phase retrieval is Fourier ptychography^118–121^. Ptychography was developed to solve the phase problem in electron diffraction measurements^122^. Fourier ptychography reconstructs high spatial resolution and large FOV phase from a series of intensity images^123,124^. The coded modulation illumination techniques can also achieve the same goal^118,125–133^. A close inspection of the diffraction integral reveals that the wavelength *λ* and propagation distance *z* always appear in pairs, which means that a change in wavelength is demonstrated with an equivalent effect as a change in propagation distance^103,134,135^. Compressive sensing or sampling framework aims to reconstruct a signal using measurements with a much smaller dimension, i.e., the measurement system is under-determined. The signal to be reconstructed can be represented as a sparse function in some encoding domain. From Eq. (7), the transmission functions of the object wave and its twin image are different, which causes the sparsity of the object in the focused plane, while the twin image is non-sparsity. It provides an idea of twin-image-free reconstruction from a single hologram by total variation regularization in the object plane^136,137^. The convolution of the Fresnel zone plate and the object under broadband illumination can derive the same pattern distribution as the in-line hologram. An ultra-thin lensless camera was designed based on deconvolution under total variation regularization^138^. The wave propagation itself is an efficient encoding scheme in compressive sensing, which enables phase retrieval without an object support mask^139^. The 3D sections of the sample can also be reconstructed from 2D holographic measurements^140,141^. For objects with sparse distribution in the real-domain, the support function with a limited area can be applied in the object constraints to achieve single-plane phase retrieval^142–146^. Sparsity-based multi-height phase retrieval has demonstrated that high-quality phase retrieval can be achieved, having a significant reduction in the number of measurements required^147,148^. Recently, single-plane phase retrieval can be achieved by sparsity optimization in its derivative domain^149,150^. Combined with compressed sensing, complex amplitude reconstruction can be realized by single pixel imaging^151^. The advantage of in-line DH is that the full detector’s bandwidth reconstruction can be realized. Off-axis DH can provide a quantitative phase reconstruction while only a maximum half bandwidth of the camera can be used. Off-axis optimization phase provides an effective initial guess to avoid stagnation and minimize the required measurements of multi-plane phase retrieval^152^.

Several factors limit the resolution, which include diffraction, pixel’s size, sensor area, and coherence of the system^153^. If the pixel size can be arbitrarily small and the coherence is perfect over a large sensor area, a diffraction-limited image can be ideally reconstructed with a maximum detectable spatial frequency of *n*_*m*_/*λ*, where *n*_*m*_ is the RI of the medium between the sample and the sensor plane, and *λ* is the illumination wavelength. The half-pitch resolution of a reconstructed image using a state-of-art image sensor chip is about one micron. To achieve resolution beyond the pixel-pitch limit, a technique called pixel super-resolution (PSR) has been employed^154–157^. In PSR, the hologram is shifted laterally in sub-pixel increments, a low-resolution (LR) hologram is captured, as shown in Fig. 6e. This relative lateral shift of the hologram can be achieved by shifting the sensor^92,157^, the sample^158^, the illumination source^154,159^, active micro-scanning^160^, or the illumination patterns^105^. Using multiple LR holograms, a high-resolution hologram can be digitally synthesized^161^. The final resolution is limited by the *NA* of the imaging system. Versatile techniques based on iterative optimization can also be applied^154,156^. These iterative methods generally solve the optimization problem to minimize a cost function such as:

8 $$O_{*}={\text{arg}}\,\min \left\{\sum _{j}{\Vert {W}_{j}O-{I}_{j}\Vert }_{p}^{q}+\alpha \cdot \gamma (O)\right\}$$

where cost function typically contains two parts: the first part uses some norm (e.g., *p* = 1 or 2 with *q* = 1 or 2, respectively) to minimize the distance between the optimal solution ${I}_{j}$ and *j* different measurements, where ${W}_{j}$ represents the digital process of shifting and down-sampling of an image and ${I}_{j}$ is LR measurement, and *O* is the object. The second part is a regularization term to maintain or regulate some desired quality in the reconstruction, for instance, smoothness (derivative)^156^, sparsity (*L*_1_-norm)^162^, or sparsity in its derivative (total variation)^149,150,155,163^, The strength of this regularization term can be balanced by the coefficient *α*. Since the transformations represented by ${W}_{j}$ are linear, this cost function is typically convex^164^, and can be optimized via convex optimization algorithms such as gradient-based and conjugate-gradient-based algorithms^156,161^. The final reconstruction from lensless on-chip imaging with an image quality comparable to a high-end conventional compound microscope has been demonstrated^92,116^, as shown in Fig. 6g.

With the help of the improvement of computing ability, neural networks (NN) can be used in holographic reconstruction and phase retrieval^165,166^. Phase retrieval of dense and connected samples is in general a challenging task that requires measurement diversity to find a robust solution. After appropriate training, a convolutional neural network (CNN) can reduce the twin image and self-interference-related noise terms. It achieves the reconstruction of biological samples using a single hologram with imaging quality comparable to multi-height phase retrieval^165^. This further simplifies lensless imaging hardware and reduces its computational load in the reconstructed process with a well-trained network. It would be the key to real-time imaging of various specimens. The general framework of the learning method is crucial for image annotation and automated detection of specific features within a microscopic image. It is also transformative for the design and implementation of mobile computational imagers, especially for telemedicine and biomedical sensing applications, among others.

### Off-axis holography and spatial multiplexing

**Fig. 7:**
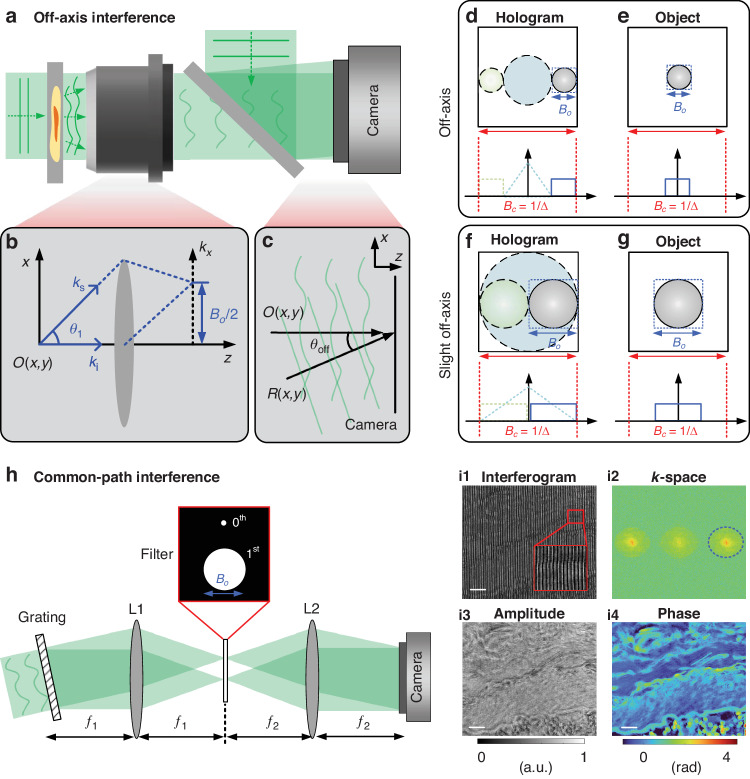
Off-axis holographic imaging model. The interferometry offers *k*-space shifting between the object field and the reference field. The spatial field distribution in the complex-domain is reconstructed from a single interferogram in the real-domain. It belongs to the Fourier-domain interferometric method in Fig. 3e. **a** Off-axis configuration, the reference field is tilted to create spatial modulation. **b** Light scattering geometry, *U* is the incident field, *k*_*i*_ is the incident wavevector and *k*_*s*_ is the scattering wave vector. **c** off-axis recording in the camera. *θ*_off_ is the off-axis angle between the object wave and reference wave, and it can be divided as *θ*_x_ and *θ*_y_ in *x* and *y* axis, respectively. **d** The FT of the off-axis interferogram and the bandwidth limitation. **e** The FT of the object wave in off-axis interference. **f** The FT of the slight off-axis interferogram and the bandwidth limitation. **g** The FT of the object wave in slight off-axis interference. **h** The configuration of common-path diffraction phase. **i1-i4** The off-axis holography, the *k*-space, the reconstructed amplitude, and the reconstructed phase

The interferometry can offer *k*-space shifting between the object wave and reference wave. The spatial complex-amplitude in the complex-domain is reconstructed from a single interferogram in the real-domain. It belongs to the Fourier-domain interferometric method in Fig. 3e. Leith and Upatnieks’s pioneering paper on off-axis holography was titled “Reconstructed Wavefronts and Communication Theory”^8^, suggesting upfront the transition from describing holography as a visualization method to a way of transmitting information. In full analogy to the methods of radio communication, off-axis holography essentially adds spatial modulation. The off-axis configuration is shown in Fig. 7a. The coherent illumination is considered in the transmission imaging system. The reference wave is introduced after the imaging system. Angular light-scattering measurements provide the spatial frequency distribution of the field, as shown in Fig. 7b. Angle-resolved intensity measurements of the scattered light depend on the object wave, which in turn yields information about the internal structure of objects, even for a weak absorption. Due to the ability to study inhomogeneous and dynamic samples through direct measurements, light-scattering methods have been used broadly, from atmospheric science to soft condensed-matter physics and biomaterials^167^. Note that the Fourier relationship between the image and scattered light only applies to complex fields, not intensities. The maximum scattering angle recording depended on the *NA* of the system, which determines the spatial resolution and produces a band-limited wave with the bandwidth ${B}_{o}$. The oblique light creates a linear phase shift, which enables the reconstruction of wavefronts from a single off-axis hologram. The tilt phase of the reference beam $R(x,y)={E}_{r}\exp [i\varphi (x,y)]$ can be represented by the following mathematical expression:

9 $$\varphi (x,y)=\frac{2\pi }{\lambda }[x\,\sin ({\theta }_{x})+y\,\sin ({\theta }_{y})]$$

where *θ*_*x*_ is the angle between the projection of the illumination direction of the reference wave on the *x*–*z* plane and the *z* axis. *θ*_*y*_ is the angle between the projection of the reference wave illumination direction on the *y*–*z* plane and the *z* axis. *λ* is the wavelength. The phase is linearly dependent on space is converted to a shifted Dirac increment function in the Fourier domain, which causes the object term to shift from the center. It can be expressed as:

10 $$\begin{array}{c}{ {\mathcal F} }(I)={ {\mathcal F} }[{|O(x,y)|}^{2}]+{|{E}_{r}|}^{2}\delta ({k}_{x},{k}_{y})+|{E}_{r}|{ {\mathcal F} }[O(x,y)]\otimes \delta [{k}_{x}+\frac{2\pi }{\lambda }\,\sin ({\theta }_{x}),{k}_{y}+\frac{2\pi }{\lambda }\,\sin ({\theta }_{y})]\\ +\,|{E}_{r}|{ {\mathcal F} }[{O}^{\ast }(x,y)]\otimes \delta [{k}_{x}-\frac{2\pi }{\lambda }\,\sin ({\theta }_{x}),{k}_{y}-\frac{2\pi }{\lambda }\,\sin ({\theta }_{y})]\end{array}$$

where $({k}_{x},{k}_{y})$ is the coordinate in the Fourier domain, and ${ {\mathcal F} }$ is 2D FT, and $\otimes$ is the convolution operator. The third and fourth terms in Eq. (10) include the object and its conjugate term, which can be regarded as a cross-correlation spatial Fourier spectrum between the object wave and reference wave. Figure 7d, e show the Fourier domain of off-axis recordings. The object’s Fourier space is completely separated from other spatial Fourier spectra. The maximum bandwidth of the object wave ${B}_{o}$ needs to be less than a quarter of the bandwidth of the detector ${B}_{c}$. The object wave can be reconstructed by filtering the object Fourier space from Fig. 7d. When the bandwidth of the object becomes lager, the slightly off-axis configuration can record the object with half of the camera’s bandwidth, as shown in Fig. 7f, g. Phase shifting by using multi-frame is needed to reconstruct the object wave due to the overlapping with the AC term. The analytical signal based on the band-limited wave can assist the reconstruction of a slightly off-axis hologram, which is introduced in the following. The advantage of off-axis methods is the single-shot capability, allowing high-speed imaging. Phase sensitivity in temporally plays a crucial role in the operation of phase reconstruction. Temporal phase sensitivity governed by the phase stability of the instrument, is the most challenging feature to achieve. Temporal phase noise is generated by the optical path length shift between the reference and object beams due to mechanical vibrations, air fluctuations, or electronic noise that occurs in the detection and digitization process. Common path approaches have exploited the central idea in phase contrast microscopy: the incident light acts as a reference field locked in phase with the scattered field, which results in intrinsic stability^81,82,168^. Figure 7h shows a schematic of the common path interference system, which can be placed at the output port of the microscope. The diffraction phase interferometer is created using a diffraction grating in conjunction with a 4f system^169,170^. In this geometry, the interferometer allows highly stable and sensitive time-resolved measurements. By using the periodic nature of the diffraction grating, multiple copies of the image are created at different angles. The zeroth and first orders create the final interferogram at the camera. Under a 4f configuration, the first lens takes a FT, creating a FP. A spatial filter is placed in the FP, which allows the full first order to pass. The zero order is filtered using a small pinhole such that the field becomes a uniform plane wave and serves as the reference wave to the interferometer after the second lens. The two fields interfere with each other on the camera to create the interferogram. These interferometers were followed by common-path interferometers where the reference beam and sample beam travel along the same path, reducing measurement sensitivity to vibration^171^. A common-path interferometer microscope built by Dyson was used to image fixed biological specimens^172^. The object’s spatial Fourier spectrum, AC term, and conjugate wave are separated, as shown in Fig. 7i1, i2. After filtering the object’s spatial Fourier spectrum, the amplitude and phase of the object wave can be reconstructed. The ability of mathematical integrity and single-shot reconstruction enable a high time–bandwidth product at the expense of the SBP. The bandwidth utilization is defined as the areas’ ratio of the cross-correlation terms to the camera’s bandwidth^173,174^. As shown in Fig. 7d, the space-bandwidth utilization for the conventional off-axis is only 9.31%, which causes a waste of sensor bandwidth and a limited FOV or resolution of reconstruction.

**Fig. 8:**
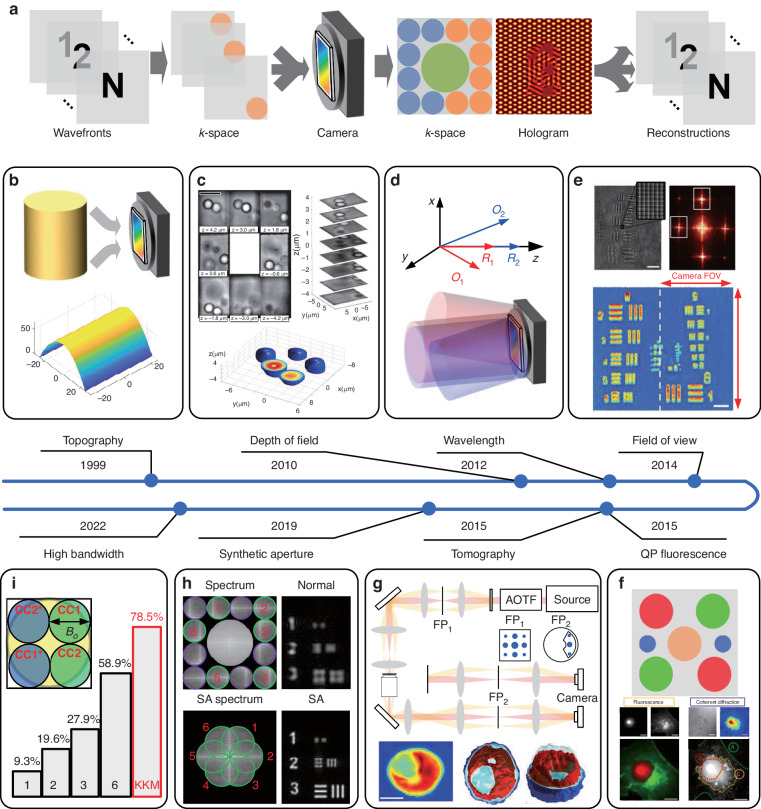
Off-axis holographic multiplexing imaging model. The multiplexing involves capturing several complex-value wavefronts in one hologram using coherent illumination, enabling data compression in void bandwidth of interferogram. **a** Schematic illustration of the holographic multiplexing concept. **b** Spatial multiplexing for 3D displacement metrology measurements^175^. **c** DOF multiplexing^182^. **d** Wavelength multiplexing, multiplexing of several off-axis holograms acquired with different illumination wavelengths^184^. **e** FOV multiplexing in a single digital camera^194,198^. **f** Multiplexed holography/fluorescent microscopy^205^. **g** Multiplexing for holographic tomography^211^. **h** Multiplexing for simultaneous synthetic-aperture super-resolution imaging^212^. **i** High bandwidth utilization holographic multiplexing framework^230^

The *k*-space of the sample can be separated from its conjugation term and the AC term in *k*-space. The separation typically occurs across a single axis, which allows the compressing of more wavefront along the other axes as well. This can be achieved by optical multiplexing of several wavefronts with different interference fringe orientations into a single hologram, known as spatial holographic multiplexing (SHM). Figure 8a presents a scheme of the entire multiplexing process. In Step 1, multiple object waves interfere with the corresponding reference wave under different interference angles, which results in different positions of carried frequency in the *k*-space. In step 2, multiple sample and reference beams are projected onto the camera. Each combination creates a straight off-axis interference fringe in a different direction, and yields a different cross-correlation pair in the *k*-space. The multiple object waves are reconstructed by filtering the corresponding spatial Fourier spectrum and performing IFT. In 1999, spatial multiplexing was applied to perform 3D displacement measurements^175^, as shown in Fig. 8b. It can be applied to perform 3D shape measurements, followed by phase unwrapping with multiple illumination angles^176–178^. Multiplexing from multiple viewpoints can be used to improve the 3D position measurements, which are typically carried out for particle tracking^179–181^. By using an SLM, several focal planes of object wave are employed for sharp multiplexing in single camera acquisition^182^, as shown in Fig. 8c. The multiplane samples can be encoded in a single camera acquisition with rejection of out-of-focus artifacts^183^. Different layers of the sample create different fringe orientations on the camera simultaneously by using a low-coherence interferometric setup, and the different layers do not interact with each other due to coherence gating.

Different object waves can also interfere with each other, which causes an unwanted cross-correlation term between object waves in the *k*-space. When multiplexing various illumination wavelengths, this unwanted term can be eliminated^184,185^, as shown in Fig. 8d. Multiplexing different wavelengths of the same sample instance can be used for both multiwavelength phase unwrapping and holographic spectroscopy. The quantitative phase retrieved is 2π periodic due to the calculation of inverse tangent. When objects are optically thicker than the wavelength, the phase is wrapped and subjected to phase measurement ambiguity. An experimental solution to the phase ambiguity problem is two-wavelength holography^186–188^. Another application of simultaneous multiwavelength holography is holographic spectroscopy and RI dispersion^189–192^. The RI and physical thickness from the phase measurement can be decoupled using multiple wavelength measurements^193^.

Multiple wavefronts can describe different spatial portions of object waves, facing the application of FOV expansion. In 2014, an external holographic module was proposed, which projected two interference patterns of different fringe orientations onto the camera^194^. Each of wavefronts captures a different area of the sample, as shown in Fig. 8e. They can be both fully reconstructed and stitched together without loss of resolution or magnification. Further extensions of the double imaging area principles have been proposed to achieve broadband holography and flipping interferometry^195–197^. The FOV holographic multiplexing approach can be extended to multiplex three FOVs of the sample into a single hologram^198^. The image of the sample is created at the output of the microscope, where the three FOV multiplexing module is located. FOV multiplexing can also be employed in common-path multi-beam interferometry with small shearing^199–202^. The hybrid amplitude cross-checker^203^ and randomly encoded^204^ gratings recently emerged as diffraction-efficient approaches. The diffraction grating profile determines the multibeam interference angles, hence enabling the tailoring in the *k*-space of the hologram.

Holography provides the quantitative phase profile without labeling but does not have molecular specificity. Multiplexing of holography with fluorescence can overcome this challenge. Simultaneous acquisition of holographic and fluorescent channels is advantageous for dynamic samples^205,206^, which requires both the QPI provided by DH and the molecular specificity provided by fluorescence. White light diffraction phase microscopes were constructed which makes it possible to multiplex two fluorescent channels and one regular off-axis holographic channel^205^. The distribution of *k*-space and the hologram are shown in Fig. 8f. The phase and fluorescence of the sample can be reconstructed simultaneously from one hologram.

Multiple wavefronts can describe different spatial frequencies of the sample, which enable synthetic-aperture phase reconstruction and holographic tomography. Optical imaging systems are restricted both axially and transversally in resolution due to the diffraction^207^, which limits the transversal resolution to a value of $\beta \lambda /NA$, where $\beta$ is a constant that depends on the imaging system configuration and it has a value of 0.82 for coherent imaging with circular apertures^208–210^. The previous equation is modified for illumination according to $\beta \lambda /(NA+N{A}_{illu})$, where $N{A}_{illu}$ relates to the *NA* of the illumination. It is also possible to increase *NA* synthetically by modulating illumination within the *NA* allowed, which provides the basement of synthetic-aperture and tomographic reconstruction^211,212^, as shown in Fig. 8g, h. For the synthetic-aperture reconstruction, the first approach regarding super-resolution using multiplexed holography is from the early 1970’s^213^. The capabilities can be extended by including a mathematical derivation and applying the approach to 2D objects^214^. In single-shot super-resolution techniques, the first paper was proposed using spatially incoherent illumination in an interferometric layout formed from diffraction gratings around 30 years ago^215^. Various super-resolution approaches by multiplexing different degrees of freedom in holography have been reported^216–219^. The first experimental realization of six-channel multiplexing by using different spatial frequencies was demonstrated in 2019^212^. An off-axis interferometric system achieved the capability of spatially multiplexing six wavefronts while using the same number of pixels needed for a single off-axis hologram, as shown in Fig. 8h. It can be further extended to the dynamic tomographic reconstruction^220^. It provides the 3D RI distribution within weakly scattering objects, which corresponds to the internal structure of cells and tissues. Regular holographic setups need to be equipped with additional hardware that enables tomography by either rotating the sample or changing the illumination^221^. The idea of applying multiplexing appears to develop independently and stems from holographic data storage concepts of angular multiplexing^222^. The tomographic reconstruction can be achieved by multiplexing based on the object rotation configuration^223–225^ and the angular multiplexing illuminations^211,226–229^. The principle of tomographic reconstruction is introduced in the following section.

The SHM assumes that all object terms are separated from the AC terms and unwanted terms, which may limit the further improvement of space bandwidth utilization of hologram. By constructing analyticity functions of multiplexing wavefronts, the full bandwidth utilization of sensor in a diffraction-limited optical system with circular-based optical transfer function can be achieved based on the KK relations^230^, realizing higher SBP on the same detector, as shown in Fig. 8i. The maximum multiplexing bandwidth utilization of 78.5% can be realized for a single hologram. When the sample is sparse, the multiplexing can be done more efficiently based on the sample sparsity^231,232^. Various mathematical tools of signal and image processing, such as wavelet compression^233–235^, can be used for SHM.

### Sideband modulation holography

The *k*-space modulations offer side-band measurement using the analyticity of sideband wave. In the non-interferometric configuration, the spatial complex-amplitude in the complex-domain is reconstructed from detections in real-domain. It belongs to the Fourier-domain non-interferometric method in Fig. 3f. The universal model of holographic can be considered as the complex-amplitude reconstruction from intensity measurement. The KK relations describe the connection between the real and imaginary parts of a complex function. To be specific, a square-integrable function $f({x})$ that is analytic in the upper half-plane of *x* satisfies the equation^236,237^:

11 $${\text{Im}}[f(x)]=-\frac{1}{\pi }p.v.{\int }_{-\infty }^{\infty }\frac{{\mathrm{Re}}[f(\tau )]}{\tau -x}d\tau$$

where p.v. indicates the Cauchy principal value. In the discrete intensity images, the contour integration directional Hilbert transform can be computed as^238^

12 $$\frac{1}{\pi }p.v.{\int }_{-\infty }^{\infty }\frac{{\mathrm{Re}}[f(\tau )]}{\tau -{x}}d\tau =i{{ {\mathcal F} }}^{-1}\{{ {\mathcal F} }\{{\mathrm{Re}}[f({x})]\}{\mathrm{sgn}}({{k}}_{x})\}$$

where ‘sgn’ is a signum function, and ${ {\mathcal F} }$ is 2D FT, and ${{ {\mathcal F} }}^{-1}$ is 2D IFT and ${{k}}_{x}$ is the coordinate in Fourier domain. The key idea is to construct an analytical description of the complex-amplitude wavefront, whose corresponding intensity has a central symmetrical spatial Fourier spectrum then it can be regarded as the real part of a complex signal. The KK relations can be applied to the real part of a complex signal. The real part of the logarithm field $E(x)$ can be subjected to the KK relations, as follows:

13 $$g(x)=\,{\mathrm{ln}}[E(x)]$$

14 $${\mathrm{Re}}[g(x)]=\,{\mathrm{ln}}|E(x)|$$

where $E(x)$ only has positive spatial frequencies for analyticity and it does not vanish in the upper half-plane. By performing Eq. (12) in Eq. (14), the imaginary part of $g(x)$ can be calculated to form the complete complex-wave of $E(x)$. For simplicity, only one dimension (1D) is considered here. The 2D case can be achieved when the 1D KK relations independently treat *x* components with different *y* values^239^. The KK relations can be embedded in interferometric-based or non-interferometric systems.

**Fig. 9:**
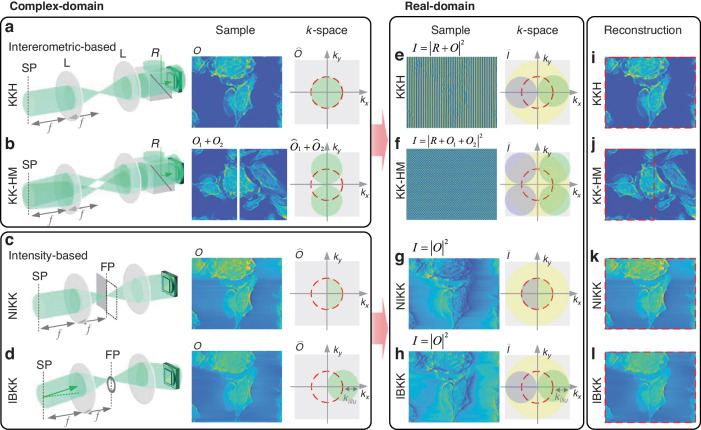
Sideband modulation holographic imaging model. The *k*-space modulations offer side-band measurement using the analyticity of sideband wave. In non-interferometric configuration, the spatial field distribution in the complex-domain is reconstructed from single detection in the real-domain. It belongs to the Fourier-domain non-interferometric method in Fig. 3f. In the interferometric configuration, it can improve the imaging throughput using analyticity of half bandwidth in the interferogram. **a** Schematic illustration of configuration, sample wave in complex-domain, and its *k*-space in the interferometric-based KK holography (KKH). **b** Schematic illustration of configuration, sample wave in complex-domain and its *k*-space in the interferometric-based KK holographic multiplexing (KK-HM). **c** Schematic illustration of configuration, sample wave in complex-domain and its *k*-space in the non-iterative intensity-based KK (NIKK) reconstruction. **d** Schematic illustration of configuration, sample wave in complex-domain and its *k*-space in the intensity-based KK (IBKK) holography. **e**−**h** The detections and its *k*-space in real-domain from (**a**−**d**). **i**−**l** The reconstructions from (**e**−**h**)

For the interferometric-based system, as shown in Fig. 9a, the conventional off-axis holography is considered and the field $E(x)={E}_{0}(x)/R(x)=1+o(x)$^43^, where ${E}_{0}(x)=O(x)+R(x)$, $O(x)$ is the sample, and $R(x)$ is the reference wave, and $o(x)=O(x)/R(x)$. The corresponding intensity and its spatial Fourier spectrum are shown in Fig. 9e. The analyticity requires only positive spatial frequencies of complex-amplitude, so the bandwidth of conventional off-axis configuration with ${B}_{s}\le 0.25{B}_{c}$ can be relaxed to half of the bandwidth ${B}_{s}\le 0.5{B}_{c}$. Slight off-axis holography can reconstruct the sample wave from one exposure, as shown in Fig. 9i. By introducing multiple sample waves, the KK relations can be embedded in SHM. As shown in Fig. 9b, the SHM with two channels is considered and the field is $E(x)={E}_{0}(x)/R(x)=1+o(x)$^230^, where ${E}_{0}(x)={O}_{1}(x)+{O}_{2}(x)+R(x)$, ${O}_{1}(x)$ and ${O}_{2}(x)$ are the sample 1 and 2, respectively, and $R(x)$ is the reference wave. The corresponding intensity and its spatial Fourier spectrum are shown in Fig. 9f. The KK relations fully utilize the sensor’s space bandwidth, allowing the AC term to overlap with the sample spectra for the multi-channel recording. In SHM, the interference not only occurs between the sample wave and reference wave, but also between different object waves. Comparsing with the conventional SHM, the KK relations circumvent the requirements that the samples’ *k*-space need to be separated from other terms. Then it improves the bandwidth utilization from 58.9% to 78.5%^230^. Only the analyticity of the multiple sample waves is required by the positive spatial frequencies of complex-amplitude in KK relations, as shown in Fig. 9f. The interferometric-based KK imaging is essentially a nonlinear filtered demodulation hologram based on Hilbert transform^240–242^. The effect of the zeroth diffraction spectrum can be eliminated.

Holographic imaging technically requires an interferometric setup, a coherent source, and long-term stability. By modulating the *k*-space of the sample wave, it is possible to achieve complex-amplitude reconstruction from single intensity measurement by using KK relations^243^. As shown in Fig. 9c, the pupil modulation ensures that the *k*-space of the sample wave meets the analyticity and the *k*-space only has positive spatial frequencies in half of the Fourier domain. In this case, the field is defined as ${\widehat{O}}_{i}({\bf{k}})=\widehat{O}({\bf{k}})P({\bf{k}})$^44^, where the $\widehat{O}({\bf{k}})$ is the FT of sample $O({\bf{k}})$, $P({\bf{k}})$ is denoted as the scanning aperture. The edge of the pupil strictly crosses the center of the *k*-space to ensure positive spatial frequencies in half of the Fourier domain. The intensity image is measured and the central symmetrical *k*-space is obtained, as shown in Fig. 9g. It can be seen that this *k*-space is corresponded to the off-axis hologram. But only a portion of the spatial Fourier spectrum can be reconstructed, as shown in Fig. 9k. The optical resolution is still limited by the coherent diffraction limit of the microscope. The illumination modulation can directly shift the CTF to scan the *k*-space, as shown in Fig. 9d. The highest spatial frequency can theoretically reach $2N{A}_{obj}/\lambda$. The intensity and its spatial Fourier spectrum are shown in Fig. 9h, and the corresponding reconstruction is shown in Fig. 9l. Multiple exposures to cover all directions of *k*-space are needed to obtain the isotropic resolution by using synthetic-aperture^45^. The 3D RI tomography can be proposed by using the first-order Born and Rytov approximations^43^. This non-interferometric method can be attributed to phase retrieval under asymmetric matching illumination and Hilbert transform^244^. For the 3D intensity stack, the Hilbert transform is equivalent to the deconvolution of the transfer function under the Rytov approximation, achieving a direct synthetic aperture for 3D RI tomography^245^.

Spatially partially coherent illumination can be introduced in the non-interferometric setup by using LED illumination^246,247^, and the reflection wide-field intensity topography measurement^248^. For band-limited imaging systems, an expanded space-bandwidth can be achieved. A deterministic transformation of intensity information into phase information can be related by modulating incident or scattered waves. This method relaxes the restriction of spatially coherent waves for illumination. It can be anticipated that the KK relations facilitate holographic imaging in optical, X-ray, and electron imaging systems and allow the investigation of complex micro- and nano-structures^249^.

## Synthetic aperture and optical diffraction tomography

**Fig. 10:**
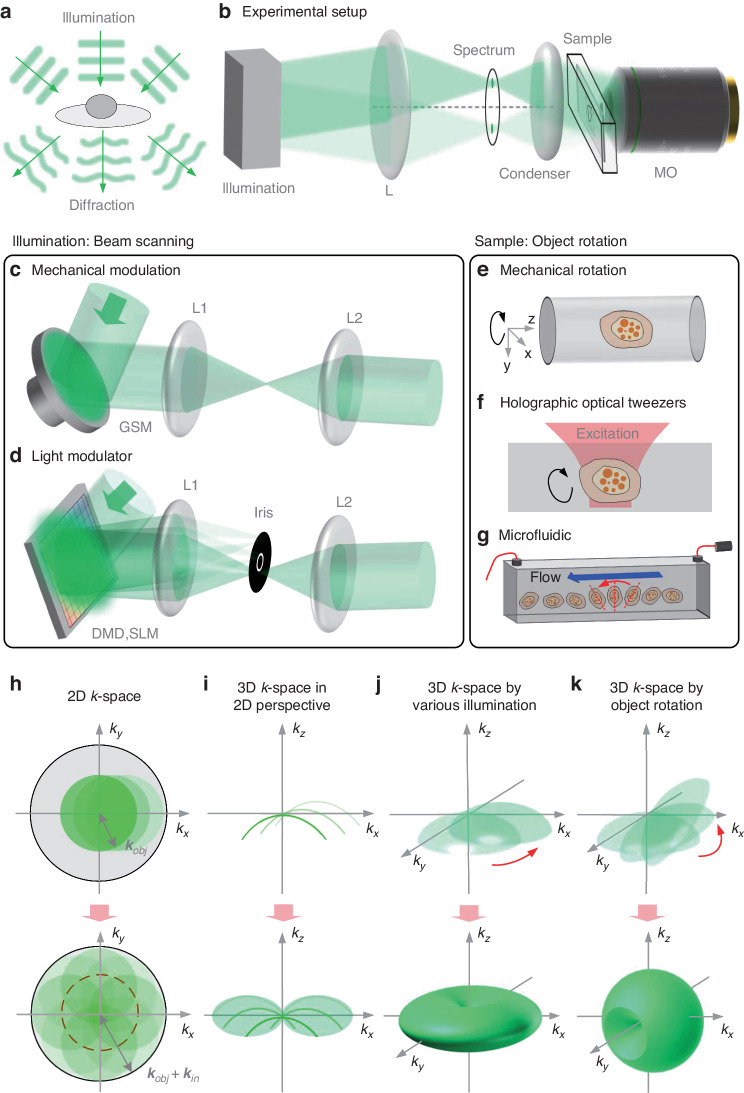
Illustration of the unified framework in diffraction tomography. Combining the spatial scanning principle of CT, the 3D RI distribution of a weakly scattering sample is reconstructed from the measurements of multiple 2-D holograms with various optical projections. The optical projections of object can be achieved by using illumination scanning and object rotations. **a** Object is illuminated by plane waves from different directions, and the total field $O({\bf{r}})$ results from the interference between the scattered field ${O}_{s}({\bf{r}})$ and the unperturbed fields ${O}_{in}({\bf{r}})$. **b** Experimental setups for standard diffraction tomography techniques. **c** Beam scanning methods based on mechanical modulation such as a dual-axis galvanometer mirror. **d** Beam scanning methods based on light modulation such as SLM or DMD. **e** Object rotation scanning methods based on mechanical rotation. **f** Object rotation scanning methods based on holographic optical tweezers. **g** Object rotation scanning methods based on microfluidic channels. **h** Supports in *k*-space for the cases of 2D imaging under angle-varied illuminations. **i** The 2D perspective of 3D supports in *k*-space for the cases of 3D imaging under angle-varied illuminations. **j** The 3D supports in *k*-space for the cases of 3D imaging under angle-varied illuminations, and the full *k*-space coverage for the scattering potential of the 3D sample. **k** The 3D optical transfer functions of the system under sample rotations, and the full *k*-space coverage for the scattering potential of the 3D sample

For QPI of 2D thin specimens, the object is described by the 2D complex-amplitude, which is $O({\bf{r}})=A({\bf{r}})\exp [i\phi ({\bf{r}})]$, where $A({\bf{r}})$ and $\phi ({\bf{r}})$ represent the absorption and phase components of the object, respectively. Similar to other imaging modalities, the resolution of DH is also limited by the coherent diffraction limit. The resolving power is limited by the wavelength and the finite aperture of the imaging system. The higher resolution can be achieved by collecting a larger angle of the diffraction wave^216,250^. For digital imaging, the pixel’s size determines the upper bound bandwidth of reconstruction. As discussed above, lensless imaging may affect the pixel’s limitation because the *NA* can be close to one. An intuitive physical method of resolution enhancement is to collect higher spatial frequency to the passband of the imaging system, as shown in Fig. 10a. The Zero-order diffraction of the object wave can be modulated by changing the angle of illumination, then higher-order diffractions can be used in the bandwidth of imaging system to improve resolution without sacrificing a wide FOV^251,252^. Inspired by the moire fringes generated by structured light illumination in fluorescence imaging^253^, structured light and speckle illumination can also encode the higher spatial frequency of object into diffraction-limit imaging system^254–258^. The nano-structure response of objects can be detected by using nonlinear effects, such as holography by second-harmonic generation^259–262^ and evanescent wave generation^263–267^. The high spatial-frequency components from the illumination modulation act on the 3D Fourier space. As developed here, 3D QPI is based on scalar diffraction. The object is assumed as weak absorption and has a 3D RI given by ${n}_{o}({\bf{r}})$. The resulting diffraction field $O({\bf{r}})$ can be described by the inhomogeneous Helmholtz wave equation:

15 $${\nabla }^{2}O({\bf{r}})+{k}^{2}({\bf{r}})O({\bf{r}})=0$$

where $k({\bf{r}})={k}_{0}{n}_{o}({\bf{r}})$, ${k}_{0}=2\pi /\lambda$ is the free-space wave vector magnitude. Using the scattering potential $v({\bf{r}})={{k}_{0}}^{2}[{n}_{o}{({\bf{r}})}^{2}-{n}_{m}]$, where ${n}_{m}$ is the average RI of the object, this becomes

16 $${\nabla }^{2}{O}_{s}({\bf{r}})+{{n}_{m}}^{2}{{k}_{0}}^{2}{O}_{s}({\bf{r}})=-v({\bf{r}})O({\bf{r}})$$

where ${O}_{s}({\bf{r}})$ is the scattered light induced by the inhomogeneous RI distribution in the object. The object’s scattering potential is reconstructed by measuring a set of diffracted intensities $I({\bf{r}})={|O({\bf{r}})|}^{2}$. The problem is to find $v({\bf{r}})$ from the measured set of intensities $I({\bf{r}})$ based on the relationship defined by Eq. (16). This is a complicated phase retrieval problem. The variable **r** represents 2D spatial coordinates $({x}{,}{y})$ for the case of 2D QPI. For the 3D case, **r** represents 3D spatial coordinates $({x}{,}{y}{,}{z})=({{\bf{r}}}_{T}{,}{z})$ with ${{\bf{r}}}_{T}$ representing the transverse spatial coordinates. Assuming that the sample is illuminated by a quasi-monochromatic plane wave with unit amplitude, the resultant total field $O({\bf{r}})$ can be regarded as the interferometric superposition of the incident field ${O}_{in}({\bf{r}})$ and the scattered field ${O}_{s}({\bf{r}})$. ODT solves an inverse problem of light scattering by a weakly scattering object. Typically, the 3D RI distribution of a weakly scattering sample or a so-called phase object is reconstructed from the measurements of multiple 2D holograms with various illumination angles, as shown in Fig. 10a. It is analogous to X-ray computed tomography (CT). Both the ODT and X-ray CT share the same governing equation – Helmholtz equation. Essentially, the optical setup for ODT consists of two parts: the illumination or sample modulation part, and the optical field recording unit, as shown in Fig. 10b. The mechanical modulation controls the angle of the illumination beam impinging onto a sample by using mirrors such as the galvanometer-based scanning mirrors (GSM), as shown in Fig. 10c. It can minimize the energy loss of illumination as much as possible. However, mechanical instability including position jittering induced by electric noise and positioning error at high voltage values may cause the nonlinear response. Without the mechanically moving part, light modulators such as SLMs^268^ and DMDs^269^ can be considered to control the illumination angles. The SLM or DMD is located at the conjugate plane of the sample. A plane wave with a desired propagating direction can be generated from the first-order diffracted beam while unwanted diffracted beams are blocked by spatial filtering, as shown in Fig. 10d. Also, the SLM or DMD can correct the wavefront distortion of an illumination beam to generate clearly plane waves. The ultra-high modulation speed reached a few tens of kHz by using DMDs. But available illumination beam power significantly decreases because of the limited diffraction efficiency and the spatial filtering. Undesired diffraction from an SLM or DMD may cause additional speckle noise or a reduction in beam power.

Instead of illumination control, a sample of interest can be rotated, and the diffracted light fields at various rotation angles can be used for the 3D RI reconstruction of the sample. The reconstructed tomograms exhibit isotropic spatial resolution along the direction of rotation. As shown in Fig. 10e, mechanical rotation of the cells enables diffraction from different projection angles^270,271^. Perturbation occurs during mechanical rotations, called radial run-out^272^, and field distortion due to the refraction from the cylindrical microcapillary requires additional numerical correcting algorithms^273^. Optical tweezers were used to rotate cells in a microfluidic channel to measure the 3D morphology of cells with precise rotation angles^274–276^, as shown in Fig. 10f. For the microfluidic, cells flow evenly through the micro-channel. The corresponding rotation angle can be calculated by the self-correlation coefficient of the single flowing cell, enabling sample rotations for 3D tomographic reconstruction^277^, as shown in Fig. 10g. The sample rotation method can cause deformation of live biological cells because of the viscoelasticity of cells, possibly resulting in artifacts in reconstructed tomograms.

The contribution of the object as a complex phase function takes the form^245^:

17 $${\phi }_{s}({\bf{r}})=\,{\mathrm{ln}}[O({\bf{r}})/{O}_{in}({\bf{r}})]$$

18 $${\mathrm{ln}}[1+{O}_{s}({\bf{r}})/{O}_{in}({\bf{r}})]=\alpha ({\bf{r}})+i\phi ({\bf{r}})$$

For the case of 2D imaging, the significance of complex phase function is apparent: ${\phi }_{s}({\bf{r}})=\alpha ({\bf{r}})+i\phi ({\bf{r}})$, where $\alpha ({\bf{r}})=\,{\mathrm{ln}}\,A({\bf{r}})=\,{\mathrm{ln}}I({\bf{r}})/2$ and $\phi ({\bf{r}})$ represent the absorption and phase parts of the sample. Under the first-order Born or Rytov approximation, a linearized relation between the first-order scattered field ${O}_{s1}({\bf{r}})={O}_{in}({\bf{r}}){\phi }_{s}({\bf{r}})$, and object function can be established for both 2D and 3D cases:

19 $$\widehat{O}({{\bf{k}}}_{T}-{{\bf{k}}}_{in})={\widehat{O}}_{s1}({{\bf{k}}}_{T})P({{\bf{k}}}_{T})$$

20 $$\widehat{O}({\bf{k}}-{{\bf{k}}}_{in})=4\pi i{k}_{z}{\widehat{O}}_{s1}({{\bf{k}}}_{T})P({{\bf{k}}}_{T})\delta ({k}_{z}-\sqrt{{{k}_{m}}^{2}-{|{{\bf{k}}}_{T}|}^{2}})$$

where **k** is the spatial frequency coordinates corresponding to **r**. For the 3D sample, ${\bf{k}}{\boldsymbol{=}}({{\bf{k}}}_{T},{k}_{z})$ is the 3D spatial frequency coordinates. ${{\bf{k}}}_{in}$ is the incident frequency vector, $\widehat{O}$ and ${\widehat{O}}_{s1}$ represent the FT of *O* and ${O}_{s1}$, respectively. *P* is the 2D complex pupil function of the imaging system, i.e., CTF, which ideally is a circ-function with a radius of *NA*_obj_/*λ*. For the case of 2D samples, the first-order scattered field gives the object’s *k*-space within a shifted pupil function, as illustrated in Fig. 10h. The phase aberrations in the reconstruction need to be considered in the coherent system^278,279^. In the 3D Fourier domain, the 3D frequency vector **k** is located on the Ewald sphere with a radius of ${{k}}_{m}={n}_{m}/\lambda$ under the constraint ${k}_{z}=\sqrt{{{k}_{m}}^{2}-{|{{\bf{k}}}_{T}|}^{2}}$. The 3D object frequency accessible by the microscope is directly related to the projection of the 2D pupil function onto a subsection of the Ewald sphere, which can be regarded as the generalized aperture $P({\bf{k}})=P({{\bf{k}}}_{T})\delta ({k}_{z}-\sqrt{{{k}_{m}}^{2}-{|{{\bf{k}}}_{T}|}^{2}})$. It is shifted by the incident frequency vector $-{{\bf{k}}}_{in}$, as shown in Fig. 10i. The Fourier diffraction theorem suggests that each measurement of a first-order scattered field ${U}_{s1}$ can only provide limited object frequency located on the shifted generalized aperture. Adjusting ${{\bf{k}}}_{in}$ enlarges the accessible object’s *k*-space, which allows the reconstruction of the scattering potential of the 3D sample. The maximum illumination angle is limited by $N{A}_{obj}$, where the maximum *k*-space coverage is the same as the incoherent diffraction limit with a doubled lateral bandwidth compared to coherent diffraction limit. The maximum *k*-space coverage forms a torus-shaped region of $\widehat{O}({\bf{k}})$ with the half-side lateral and axial extensions of the frequency supports given by $2N{A}_{obj}/\lambda$ and $({n}_{m}-\sqrt{{{n}_{m}}^{2}-N{{A}_{obj}}^{2}})/\lambda$, as illustrated in Fig. 10i, j. However, the smaller extension along ${k}_{z}$ direction (optical axis) so-called “missing cone” translates into a lower axial resolution, and limits sectioning capabilities^280^, because the diffraction wave under a 90-degree illumination angle is almost impossible to be recorded, as shown in Fig. 10j. Another approach of rotating the sample can obtain an almost complete spherical support, as shown in Fig. 10k. A small set of missing frequencies still exist along the rotation axis^281^ (i.e., *y* axis in the example), slightly degrading the resolution in this direction^282^. To simultaneously obtain improved and isotropic resolution, one can combine the illumination modulation and specimen rotations^283,284^. For samples presenting high RI, this technique could also benefit from advanced numerical reconstruction methods to allow super-resolution in far-field microscopy^285^. For thick samples, optical imaging only achieves short imaging depth owing to multiple scattering and sample-induced aberration, caused by the inhomogeneity of the RI of samples. To reconstruct more accurate forward propagation models, recent studies have exploited such as a multi-slice-based method^286,287^, learning-based method^288–290^, modified Born series^291^, convolution approach of the Lippmann–Schwinger equation^292^, etc. The high-order scattering models can help to analyze subcellular morphological changes inside thick specimens and estimate biologically relevant information, as well as the vector optical characteristics such as polarization^293,294^, birefringence^295^, etc.

## Reflection quantitative phase imaging

**Fig. 11:**
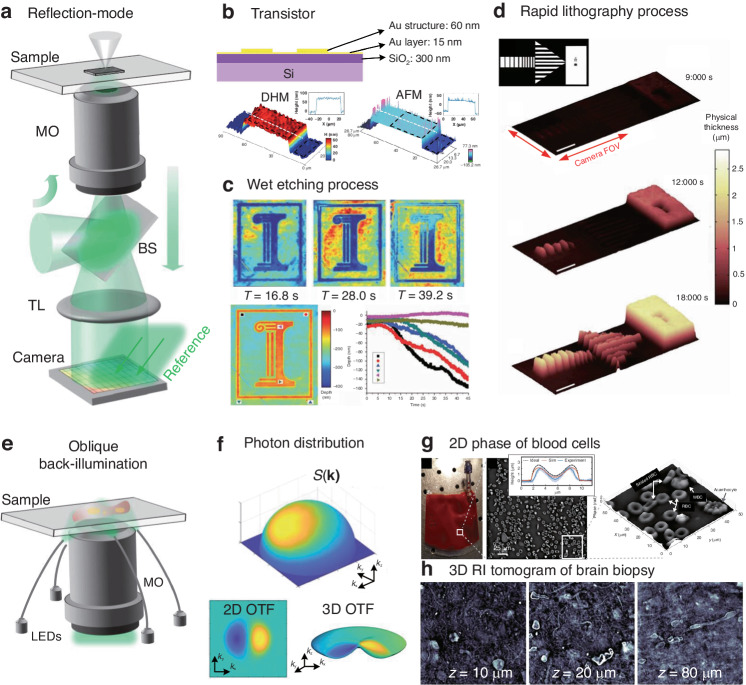
Reflection-mode phase reconstruction. Holographic imaging can reconstruct the complex-amplitude of reflection waves, enabling to detect the profiles and topography of optical opaque objects. **a** DHM setup operating in reflection mode. **b** Design of the gold electrodes in transistor sample, and its eight maps of the sample from the refection-mode DHM system and atomic force microscopy system^298^. **c** Real-time imaging of the wet etching process^299^. **d** Quantitative thickness imaging of a rapid lithography process^194^. **e** LED-illuminated inverted quantitative oblique back-illumination microscopic configuration^305,306^. **f** Photon angular distribution collected at the sample plane, the corresponding 2D phase transfer function in pupil plane coordinates and the 3D optical transfer function distribution in the 3D pupil plane coordinates. OTF: Optical transfer function. **g** The reconstructed phase image of blood cells in the bag from a patient with sickle-cell disease^305^. **h** 3D slice of RI tomogram of brain biopsy^306^

The transparent objects show weak absorption of illumination, and the distribution of its inner RI and thickness scattering samples are contained in the light scattering and they convert into phase distribution of wavefront. We can measure the complex-amplitude of transmitted wavefront to reveal the 2D and 3D structure of transparent objects. For the reflective wavefront, the light is bouncing back and does not passing through the opaque object. The optical path delay in different areas describes the topography of the opaque object, which can be reconstructed by the phase using holographic reconstruction operating in reflection mode^296,297^. The only difference between reflection and transmission mode is in the light path within the microscope, as shown in Fig. 11a. In reflection mode, the collimated beam entering the back of the microscope passes through a collector lens and is focused onto the back focal plane of the microscopic objective. The objective lens then creates a collimated beam in the sample plane. The scattered and unscattered waves reflected by the sample are collected by the objective and focused in its back focal plane again. A beam splitter redirects the light through a tube lens (TL), creating a collimated beam containing the image at the output image plane of the microscope. The contributions to the phase of total fields include double-transmitted light, backscattered light, and multi-scattered back-propagating light^22^. The phase map is converted to the height map, with the following transformations for transmission mode and reflection mode^169^, respectively:

21 $$h(x,y)=\frac{\phi (x,y)\lambda }{2\pi \Delta {n}_{0}}$$

22 $$h(x,y)=\frac{\phi (x,y)\lambda }{4\pi {n}_{m}}$$

where $\phi (x,y)$ is the phase map, and *λ* is the wavelength of the laser source. For transmission mode height calculation using Eq. (21), $\varDelta {n}_{o}$ is the RI contrast between the sample and the medium. For reflection mode height calculation using Eq. (22), as light first travels in the air to the surface of the sample and then gets reflected, there is a factor of two, which accounts for the double-pass, i.e., $\varDelta {n}_{o}$ is replaced with 2, and ${n}_{m}$ is the RI of the surrounding medium. The phase in reflection mode can quantitatively reveal the height map of the fabricated gold electrodes by depositing Au patterns on Si/SiO_2_ substrate^298^, as shown in Fig. 11b. An Au structure with a thickness of 60 nm was deposited by thermal deposition and photolithography. Then, another 15 nm Au layer was deposited over the whole device to make the sample fully reflective. Through this full-field label-free height reconstruction, the dynamics monitoring of the wet etching process in real-time can be realized. These sensitivity and accuracy results underscore the capability of reflection mode holography to monitor dynamic topographic changes at the nanoscale. The topography of an n^+^ GaAs wafer while being etched with a solution of H_3_PO_4_:H_2_O_2_:H_2_O is presented by using reflection mode holography^299^, as shown in Fig. 11c. The etch rate maps across the structure at different moments during the process and the final etched structure are presented by the height map from the quantitative phase. The time-resolved etch depth profile at discrete points across the sample can be visualized by real-time monitoring. The reflection mode to image through transparent viewing windows of semiconductor manufacturing tools enables engineers to better monitor and control the performance properties of the fabricated micro- and nano-devices. Quantitative thickness during the rapid generation of thin elements by projection photolithography can also be revealed by the reflection mode holography. Figure 11d shows the optical thickness profiles of the element during the curing of the photoresist at different time points^194^. This technique has demonstrated the ability to monitor wet etching in situ with nanometer accuracy. This method could potentially be used to create a multi-user cleanroom tool for wet etching that would allow the user to observe all this information during etching. It could greatly improve the quality of the etch as well as the overall yield. Furthermore, the ability to accurately measure these inhomogeneities in real time may provide the means to correct the fabrication instantly by perfusing the etching chamber with an adjustable etchant concentration^299^.

Methods for QPI typically involve interfering beams of a coherent source^300^. Phase contrast can be produced simply with partially coherent asymmetric illumination in a typical wide-field microscope^301^. Images produced from incoherent or partially coherent light have the advantage of resolution enhancement and a lack of speckle or other coherent artifacts^302^. The phase contrast produced from asymmetric illumination can be used to reconstruct the phase with a complete linearized model via a regularized deconvolution with the optical transfer function of the microscope^303^. Using principles of asymmetric illumination to recover the quantitative phase, it provides the possibility of the reflection mode without interference, as shown in Fig. 11e. It is also named epi-mode configuration. The prefix epi stands for epi-illumination and stems from the fact that the light is collected from the same side of the sample where it is incident. With an overall oblique illumination at the focal plane, lateral variations in the index of refraction redirect light toward or away from the acceptance angles of the objective’s *NA*, producing phase contrast in observed intensity^304^. From the distribution obtained from the photon transport simulation for the light source, an optical transfer function can be produced for the differential phase contrast image formed by the microscope, as shown in Fig. 11f, which can be applied to reconstruct the object with deconvolution^305^. The phase of whole blood from a patient with sickle cell disease inside a clear blood collection bag is reconstructed from this non-interference reflection mode holography, as shown in Fig. 11g. The normal biconcave and sickled RBC, acanthocytes, and white blood cells along with their internal contents including the nucleus can be revealed. In addition to extracting feature-rich morphology, it maintains a reliable quantitative phase profile. By using the principle of KK relations, accurate reconstruction of the surface topography can be achieved in a single shot by combining holographic imaging and spectral multiplexing when the angles of incidence precisely match the *NA* of the optical system^248^. An analysis of the 3D system is considered for a more complete reconstruction of the RI distribution of thick objects. By deconvolution with a 3D transfer function, this method can effectively remove contributions from out-of-focus planes and yields high-resolution tomogram in thick specimens from a stack of 2D images. Example 3D optical transfer function distribution in 3D pupil is shown in Fig. 11f. A full-field image of a portion of unaltered fresh human cortex tissue discarded from surgery at 48 µm of depth can be reconstructed by using epi-mode^306^, as shown in Fig. 11h. The advantages provided by reflection mode phase imaging pave the way for broader usage of 3D RI tomography, particularly for in vivo, translational, and/or clinical applications. Indeed, reflection-mode holography can provide clear visualization and quantification of axons, neuron soma, blood vessels, and other brain structures in a label-free manner. It is vitally important for improving the understanding of brain function and tracking changes that may occur in the progression of disease. It is expected that reflection-mode holography could become an important tool in the hands of researchers and clinicians alike.

## Deep holography

**Fig. 12:**
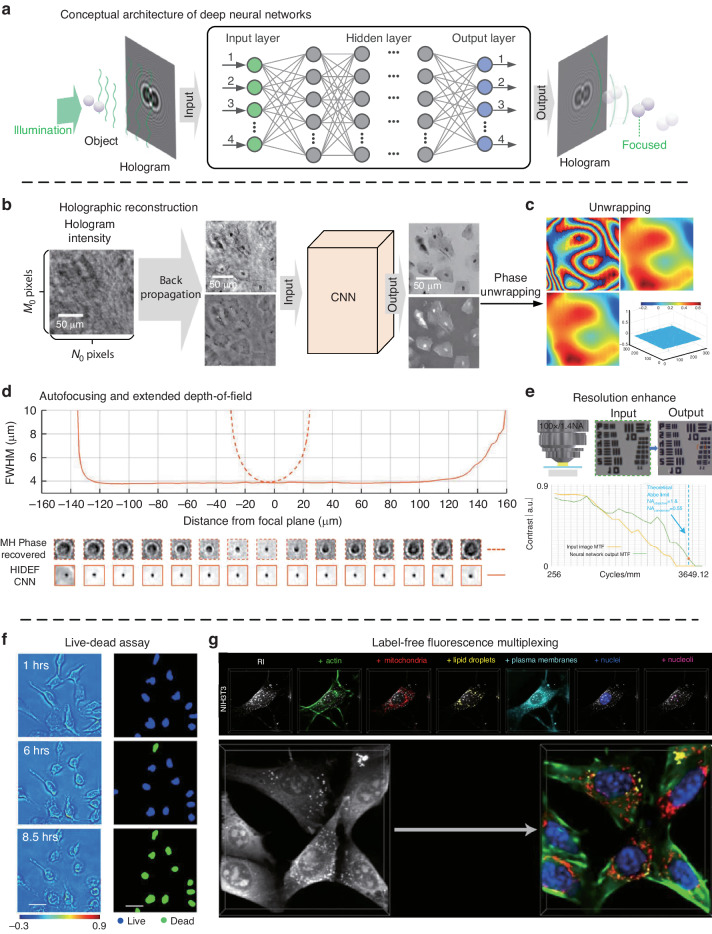
Learning-based holography. With the explosive growth of mathematical optimization and computing hardware, deep learning has become a tremendously powerful tool to solve challenging problems in holographic imaging, including every key process of reconstruction. **a** A conceptual architecture of DNN in DH. **b** The DNN architecture for holographic reconstruction^165^, the input is the reconstructed amplitude and phase from back propagation, and the output is the guess amplitude and phase of the object. **c** Phase unwrapping using DNN^327^, the compared results are performed using modified Goldstein’s algorithm^337^. **d** The CNN-based approach simultaneously performs autofocusing and phase recovery to significantly extend the DOF and the reconstruction speed in holographic imaging^331^. **e** The DNN trained for microscopic imaging, and MTF comparison for the input image and the output image of a DNN that is trained on images of a lung tissue section^332^. **f** Viability states of the individual cells from the reconstructed phase of it based on DNN^347^. **g** Label-free fluorescence multiplexing of multiple subcellular structures from RI distribution of cell^348^

Physically, holography is a two-stage process: the recording of a wavefront, and the reconstruction. Nowadays, both these processes can be performed either optically or digitally. In the past few years, we have indeed witnessed rapid progress in high-level artificial intelligence (AI), where deep representations based on NN models are learned directly from the captured data to solve various tasks. With the recent prosperous development of optimization tools called deep neural networks (DNN)^307,308^, we have witnessed the emergence of a new paradigm of solving inverse problems in various fields of optics and photonics by using DNN^309–312^. DNN can be regarded as a category of machine learning algorithms that are designed to extract information from raw data and represent it in some sort of model^308^. It provides a feasible solution to resolve the issues such as error accumulation, high computational time, and noise sensitivity that conventional techniques frequently encounter. This shift of paradigm also has a significant influence on the field of holography^313–315^. In addition to holographic reconstruction^165,316–322^, DNN has also been proposed for phase aberration compensation^323^, focus prediction^324–326^, phase unwrapping^327–330^, extension of DOF^331^, resolution enhancement^332^, and speckle reduction^333–335^. Specifically, the network is built on a collection of connected units called artificial neurons, which are typically organized in layers, an idea somehow inspired by the biological neuron in the mammalian brain. As schematically shown in Fig. 12a, a modern network consists of three kinds of layers: the input layer, the output layer, and the hidden layers. The input layer usually represents the hologram to be processed and the output layer represents the expected reconstruction that one wishes the network to produce. Data processing is mainly performed by the hidden layers between the input and the output layers. The holographic reconstruction can be treated as solving an ill-posed inverse problem for the function *G* that maps directly the hologram to the object:

23 $${G}_{learn}=\mathop{{\rm{arg}}\,\min }\limits_{{G}_{\alpha },\alpha \in M}\left\{\mathop{\sum }\limits_{n=1}^{N}L[{O}_{n},{G}_{\alpha }({I}_{n})]+\psi (\alpha )\right\}$$

where *α* is an explicit setting of the network parameters *M*, $L(.)$ is the loss function to measure the error between the *n*^th^ object ${O}_{n}$, and the corresponding in-line hologram ${G}_{\alpha }({I}_{n})$, ${G}_{\alpha}$ is the propagation operator and $\psi (\alpha )$ is a regularizer on the parameters to avoid overfitting^336^. To reconstruct both the intensity and phase simultaneously, a Y-shaped architecture is proposed for reconstruction^175^. The loss function is defined as $L(.)={L}_{I}(.)+{L}_{P}(.)$, where ${L}_{I}(.)$ and ${L}_{P}(.)$ denote the loss function of the intensity and phase of the complex wavefront. Another approach is the physics fusion or single-pass physics-informed DNN^165^, as shown in Fig. 12b. The network was trained by the reconstructed amplitude and phase using numerical propagation and the corresponding ground truths, which are reconstructed by using phase retrieval algorithms from multiple holograms^101^. This method can be applied to off-axis DH to improve the quality of the reconstructed image as well^318^. After reconstruction, the phases are usually wrapped owing to the phase ambiguities and thus need unwrapping, which is also a typical ill-posed inverse problem. Conventional phase unwrapping techniques estimate the phase either by integrating through the confined path or by minimizing the energy function between the wrapped phase and the approximated true phase^329^. An efficient segmentation network has been demonstrated to transfer phase unwrapping into a multi-class classification problem^327^, and the reconstructions are shown in Fig. 12c. The first row represents the wrapped phase and unwrapping phase by using networks. The second row shows the reconstructed unwrapped phases by modifying Goldstein’s algorithm^337^. The current networks were trained with continuous wrapped phase maps which are typical cases in interferometric optical metrology. Autofocusing is the automatic determination of the numerical calculation about the propagation distance from the hologram plane^338^. This is important for the applications in industrial and biological inspection^339^. Conventionally, the focused distance is determined by a criterion function concerning the reconstructed distance. The criterion function can be defined in various ways, such as the entropy of the reconstructed image, the magnitude differential^338^, and sparsity^340,341^. They usually have a local maximum or minimum value at the focal plane. The prediction of the focusing distance is made by directly analyzing the hologram by using a DNN. The focusing distance can be taken as an uncertain parameter to optimize automatically^342^. In this case, the objective function can be written as

24 $$[{G}_{\alpha },d]=\mathop{{\rm{arg}}\,\min }\limits_{\alpha \in M,d}\left\{\mathop{\sum }\limits_{n=1}^{N}L\{H[{G}_{\alpha }(I),d],I\}\right\}$$

where the uncertain focusing distance *d* enters the physical model *H*, and is optimized by the network. The regression approach is to train the network by using a stack of artifact-free reconstructed images that are paired up with corresponding holograms^324,343^. Taking advantage of the U-Net^344^ and ResNet^345^, the DOF in holography can be extended by using deep-learning-based autofocusing and phase retrieval^331^. In addition to bringing all the particles contained in a single hologram to a sharp focus, the network also performed phase retrieval in a long-focused distance, as shown in Fig. 12d. For the DHM, another significant performance is the spatial resolution. This expectation performance can also be realized through a deep learning approach. Various tissue samples were imaged with LR and wide-field systems, which is the input of the network. The network rapidly outputs an image with higher resolution, matching the performance of higher *NA* lenses and significantly surpassing their limited FOV and DOF^332^, as shown in Fig. 12e. The target was imaged using a 100 × ∕1.4 *NA* objective lens with a 0.55 *NA* condenser. The objective lens was oil-immersed, while the interface between the resolution test target and the sample cover glass was not oil-immersed, leading to an effective *NA* of ≤1 and a lateral diffraction-limited resolution of ≥0.355 μm. The modulation transfer function (MTF) was evaluated by calculating the contrast of different elements in the resolution test target. By using the learning method, the MTF can be extended by comparing it with conventional microscopy. It has demonstrated how deep learning significantly improves imaging resolution, FOV, and DOF.

The propagation of reconstructed wavefront from holography is described by the complex amplitude. After the computing performance is enhanced from the intensity-value measurement in the real-domain, the final significance is the expression of wavefront related to the sample and physiological activity. The existing methods to evaluate cell viability commonly require mixing a population of cells with reagents to convert a substrate to a colored or fluorescent product. But the step of exogenous staining makes these methods undesirable for rapid, non-destructive, and long-term investigation. The rapid viability assay can be conducted in a label-free manner by assisting with the learning-based method. With fluorescence-based semantic maps as ground truth, a DNN was trained to assign “live”, “dead”, or background labels to pixels in the input phase images. A U-Net based on EfficientNet (E-U-Net) is applied in the training process^346^. As shown in Fig. 12f, the viability predictions from the phase measurements of HeLa cells are obtained from the training network^347^. It suggests that the rules out the adverse effect on cell function caused by the exogenous staining, which is beneficial for the unbiased assessment of cellular activity over a long time. Based on the label-free computational specificity based on the phase, the projection of specificity from phase or RI to fluorescence specificity can be achieved by learning networks. The diffraction-limited 3D RI tomogram enables the scalable inference of 3D fluorescence tomogram of its corresponding sub-cellular targets by using data-driven learning technology^348^, named RI2FL. A large-scale dataset consisting of ~1,600 3D RI tomograms and the corresponding 3D fluorescence tomograms from 6 subcellular targets (actin, mitochondria, lipid droplets, plasma membranes, nuclei, and nucleoli) and 6 eukaryotic cell types (NIH3T3, COS-7, HEK293, HeLa, MDA-MB-231 and astrocytes) were created. All the live cell images were obtained by using standardized tomographic microscopes equipped with fluorescence channels^349^. The inferred endogenous subcellular structures and dynamics can be characterized from 3D RI tomograms by learning approach, as shown in Fig. 12g. Multiplexed tomography with RI2FL in intact living cells enables the time-resolved profiling of single-cell phenotypes. Here, we briefly describe how holography and DNNs can benefit from each other. By assisting with laser scanning confocal microscopy, CNN can translate the phase into the confocal fluorescence image^350^, providing quantitative, dynamic data, non-destructively from thick samples. It provides complementary information to that from other laser scanning techniques, and the acquisition is not limited by photobleaching and toxicity, while the axial resolution is maintained at confocal levels. For holography-inspired DNNs, most of the studies published so far focus on optical inference. The capability of light-speed processing in parallel holographic networks indeed guarantees tremendous inference power^351^.

It is also well-known that DNN has a black-box issue^352^. When it is used to solve real-world physical problems, DNN has met with limited success due to several reasons: First, DNN requires a large amount of labeled data for training, which is not always available in real application settings^353^. Physical fusion is the most straightforward way^354^. The parameterized physical forward model can impart adaptability and reliability to the deep learning model in solving the inverse problem of image retrieval^355^. A physics-enhanced DNN (PhysenNet) employs a strategy of incorporating a physical imaging model into a conventional DNN^316^. It can extend to other imaging models, such as single-pixel imaging^356^, ghost imaging^357^, and coherent diffraction imaging^358^. It is a cross-disciplinary that requires holography, neural computing, and many others. The appearance of them stimulates the development of each other and gives us a fantastic field to explore. Perhaps it is also possible to take advantage of both the physics-informed and conventional data-driven methods so that the training can be more efficient and the generalization. One can have an impression of the evolution of DNN from feedforward NN to CNN and U-Net, and so on. Neural computing provides us with more powerful algorithms, such as the spiking neurosynaptic networks that can model the behavior and learning potential of the brain^359^. The applicability and potential of these new algorithms in holography are still an open question.

## Modern application in holographic microscopy

### Holography of single-cell analysis

**Fig. 13:**
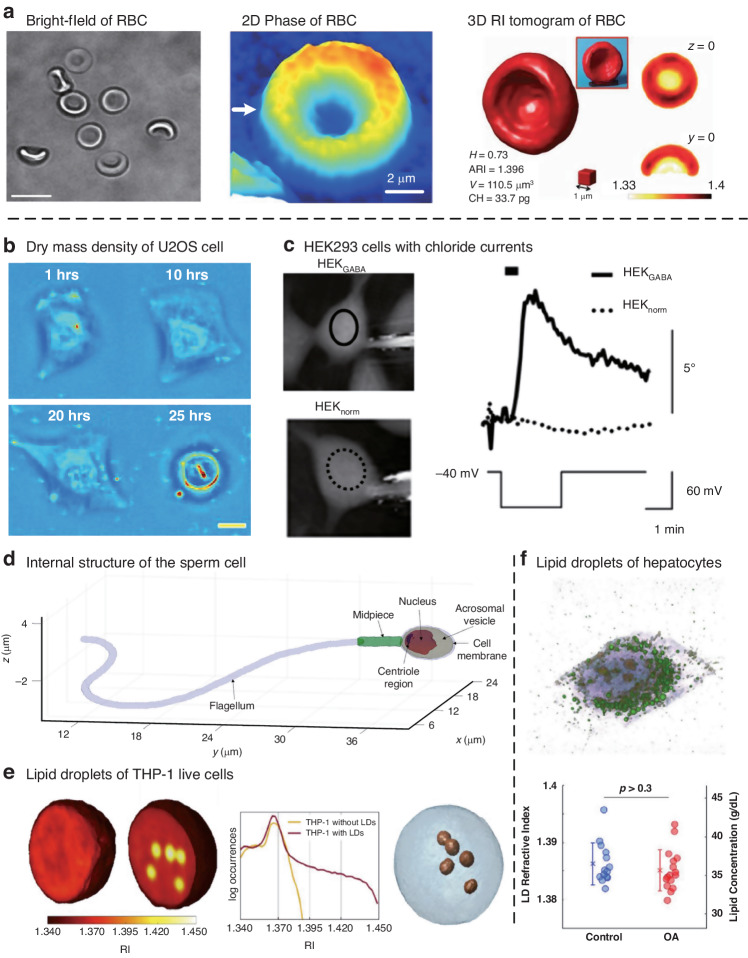
Illustration of the single-cell analysis based on holography. The physical shape and physicochemical properties of a single cell can be quantitatively reconstructed by holographic imaging, revealing single-cell resolution of subcellular structure behavior and transport. **a** The imaging of red blood cells by using bright-field microscopy^360^, phase^361^, and 3D RI tomogram^277,362^. **b** The U2OS growth over 2 days and the corresponding dry mass^369^. **c** Phase image of patched HEKGABA (left) and HEKnorm (right), application of GABA (3 mM, 30 s; bar) during a pulse of voltage^370^. **d** The 3D internal structure of the sperm cell. Light purple indicates the cell membrane (1.355 ≤ RI < 1.37), green indicates the midpiece (RI = 1.383), yellow indicates the acrosomal vesicle (1.37 ≤ RI < 1.425), red indicates the nucleus (1.425 ≤ RI < 1.465), and dark purple indicates RI ≥ 1.465 (centriole region)^371^. **e** Segmentation of the LDs within the 3D RI tomograms of THP-1 live cells^372^. **f** Quantitative analysis for LD formation in hepatocytes under oleic acid treatment^373^

Due to the tremendous progress in instrumentation development, holographic technologies are sufficiently robust to be employed in in-depth biomedical studies. One of the significant demonstrations is based on the utility of optical phase for sensing cell structure and dynamics at nanoscale. The propagation of wavefront is described by the complex amplitude. The complex-value in the complex-domain is reconstructed from the intensity-value measurement in the real-domain. The scattering wavefront from the sample produces another dimension to understand the cell structure. Figure 13a shows the bright-field imaging of red blood cells (RBC) using conventional microscopy^360^. Using phase reconstruction, the optical path delay of the cell reveals the distribution characteristics of internal material^361^. As RBCs have distinct biconcave morphology without sub-cellular organelles, 2D QPI is suited to investigate their biophysical and pathophysiological properties. The 3D RI tomograms of the human RBC have a donut-like shape^277,362^. The RBC indices can be quantitatively measured at the individual cell level. Morphological parameters such as cell volume, surface area, and sphericity are retrieved from the 3D RI of RBC. Cytoplasmic Hb concentration is directly converted from measured RI values, and Hb contents can be calculated from Hb concentration and cell volume. The RBC cytoplasm mainly consists of Hb solution without a nucleus or other subcellular organelles. Each growth pattern carries its biological significance: if the growth is linear, cells do not need machinery to maintain homeostasis. However, exponential growth requires checkpoints and regulatory systems to maintain a constant size distribution^363^. This debate has persisted despite decades of effort primarily due to the lack of quantitative methods to measure cell mass^364^. A unified, easy-to-use methodology to measure the growth rate of individual adherent cells of various sizes has been lacking. Since the RI is linearly proportional to cell density, the unique ability to reveal weight cells can be achieved by QPI. Irrespective of the constitutive molecular species, the cell phase map is measured with respect to just the culture medium. The quantitative phase yields the ‘dry mass’ density map of the cellular structure. That is the density of the non-aqueous content of the cell, which are mainly proteins and lipids^365^. Direct measurement for cell dry mass can reveal the growth of a single cell at each stage of the cell cycle. The ability to measure accurately the growth rate of single cells has been the main obstacle in answering this question^366–368^. As shown in Fig. 13b, dry mass density maps of a single U2OS cell over its entire time cycle are indicated^369^. The consistent growth of the cells and the expected 24-h cell cycle is a testament to the overall health of the culture. The results show that the mean cell mass evolves synchronously in time with the total mass of the entire population during the duration of the cell cycle. The sensitivity to nanoscale changes in thickness was employed to study live neurons during electrical activity^370^, as shown in Fig. 13c. The assistant analysis of phase makes it amenable to use for pharmacological screenings developed for the management of human pathologies involving dysfunctions of chloride channels. Perhaps one of the most impactful applications of QPI to date is non-destructively measuring single cell volume and mass over arbitrary periods in both adherent and flowing cell populations. Figure 13d shows a single frame from the 3D motion revealing the internal structure of the sperm cell^371^. The internal structures of the sperm cell are revealed from different ranges of RI. It is also expected that the RI is used to assess the male fertility of the patient’s live sperm, providing analysis of both the fine-detailed 3D morphology and motility of the sperm on an individual cell basis. The complete characterization in terms of number, size, spatial positioning, and relative distribution in the cell volume of dynamic organelles can reveal the roles in the physiological activities of normal cells or tissue. Figure 13e shows the lipid droplets (LDs) characterization of ovarian cancer cells and monocyte cell lines^372^. The RI of a monocyte with and without LDs are respectively shown, while the central slice of corresponding 3D RI tomograms was reported. The change in morphological and biochemical parameters of LDs under oleic acid (OA) treatment can be measured from the reconstructed 3D RI distributions inside the hepatocytes^373^. The results imply that the OA treatment does not affect the dry mass density of the cytoplasm and LDs. Also, QPI was used for long‑term assessments of bacteria^374^, distinguishing bacteria from sulfur mineral grains^375^, and analysis of the size of planktons^376^. Recently, the RI reconstruction can identify the cell nucleus within the label-free tomograms by using a novel computational segmentation method based on statistical inference^377^. Such a tool could revolutionize cancer diagnosis through the search for circulating tumor cells in the liquid biopsy paradigm^378–381^.

### Clinical diagnostic tool to identify healthy versus diseased states

**Fig. 14:**
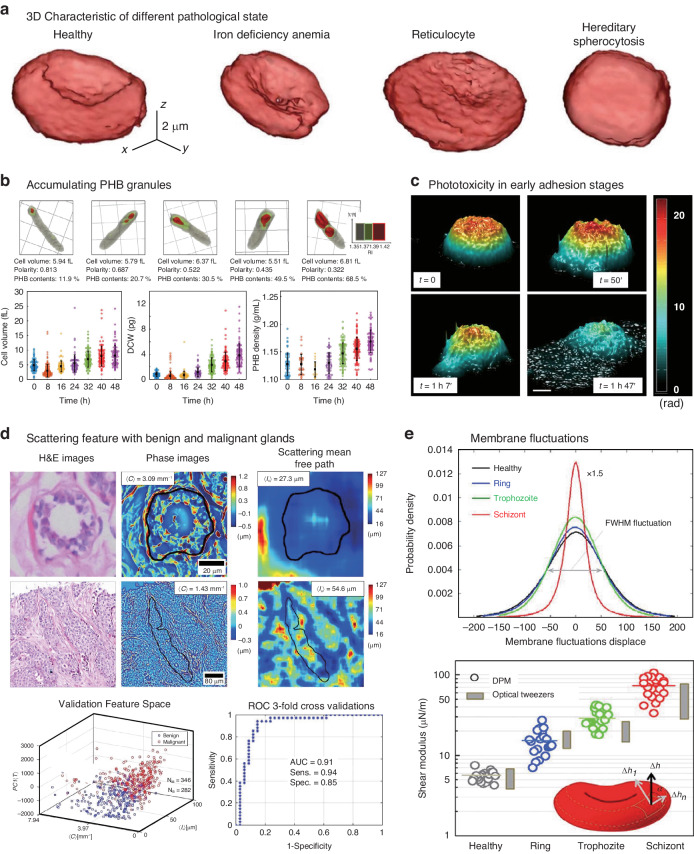
Progress toward holography clinical applications as a diagnostic tool to identify healthy versus diseased states. Holographic imaging can be applied in clinical diagnostic tools based on the physicochemical properties from the 2D phase or 3D RI reconstruction. **a** 3D rendered RI map of individual RBCs from healthy, iron deficiency anemia, reticulocyte, and hereditary spherocytosis red blood cells^387^. **b** 3D rendering images of E. coli cells with different levels of PHB accumulation, and change of time with the cell volume, dry cell weight, and PHB contents^390^. **c** Temporal evolution of the reconstructed phase map in the case of a single cell during “injurious” light exposure with phototoxicity, and the process of cell volume and cell area over time changes^391^. **d** identification of benign (top row) versus malignant (bottom row) glandular tissue, validated by pathological classification of hematoxylin and eosin-stained biopsy material, and separation of benign and malignant gland feature vectors and phase features^392^. **e** Membrane fluctuations and in-plane shear modulus at different intraerythrocytic stages of P. falciparum-invaded human RBCs^393^

There are a growing number of emerging applications for holography in clinical studies based on quantitative and label-free measurements of individual cells and cell clusters^382,383^, which provides significant advantages. Current work is mainly at the level of technology development, applications, and validation stages. The range of applications includes evaluating mitotic inhibitors with different mechanisms of action^384^, examining the regrowth rate and extent of cancer cells following senescence induction^385^, and uncovering the response heterogeneity of a mixed sensitive and resistant cancer cell population to specific drug treatment^386^. Because QPI can track the kinetics of dry mass growth responses in individual cells or clusters, where the heterogeneous cell responses to therapeutics are readily identified. Figure 14a shows the 3D RI tomograms of individual RBCs with four different pathophysiological conditions^387^, a healthy individual, a patient with iron deficiency anemia (IDA), a patient with a high reticulocyte count, and a patient diagnosed with hereditary spherocytosis (HS). The reconstructed morphologies from holographic tomography exhibit well agreement with the known reference ranges^388^. Different shapes can be visible from the tomogram. The RI tomograms of RBCs from the healthy individual exhibit the characteristic biconcave shape. The RBC from the patient with IDA decreases in the cell volume, whereas those from the patient with high reticulocyte content display a significant increase in mean volume. The RBC from the patient diagnosed with HS exhibits a spherocytosis shape. The phase can further quantify the progressive alterations to RBC membrane fluctuations and mechanical response with parasitism by P. falciparum. When combined with dry mass measurements, QPI identified and classified different kinetic states for a population of melanoma cells in culture^389^. The reconstruction of phase and RI can be used to quantify the substance accumulation of individual cell. Figure 14b shows the quantification of individual recombinant E. coli cells accumulating poly (3-hydroxybutyrate) (PHB) granules^390^. After the initial 8 h of culture, the average volumes and dry mass of the recombinant E. coli cells continued to increase. The change in the dry cell weight is more remarkable compared to the change in cell volume because the PHB granule weights were rapidly increased after 16 h. Figure 14c shows the temporal evolution of the reconstructed phase in case of a single cell during “injurious” light exposure with phototoxicity^391^. Conversely, the cell motion and vibrations appear to rise significantly when switching to “injurious” light, corresponding to the early stages of necrosis. The cell tries to regulate its volume desperately till death, as shown in the process of cell volume and area over time changes. The holographic technology can assist strain development by monitoring the detailed behavior of accumulating substance granules according to different genetic modifications and fermentation conditions. Figure 14d shows an example of phase reconstruction as a diagnostic tool for the identification of benign versus malignant glandular tissue^392^, validated by pathological classification of hematoxylin and eosin (H&E) stained biopsy material (left column). The tissue spatial correlation analysis was used to assess breast cancer fixed tissue microarrays and showed a 94% sensitivity and 85% specificity for cancer detection independent of tissue staining quality^392^. The receiver operating characteristic plot of the true positive (sensitivity) versus false positive (specificity) rate determining malignancy is from H&E counter-stained tissue biopsy. It showed the separation between benign and malignant glands in the validation feature space. Changes in dry cell mass can be used to measure single tumor cell sensitivity to activity. Figure 14e shows a histogram with membrane displacements for all parasite stages and in-plane shear modulus at different intraerythrocytic stages^393^. This histogram was then translated into the cell thickness profile by considering the optical homogeneity of the internal cell composition along the optical path. The in-plane shear modulus of the RBC membrane with attached spectral cytoskeleton could be determined from the phase by using the Fourier-transformed Hamiltonian (strain energy) and equipartition theorem^394^. In-plane shear modulus of the RBC membrane versus developmental stage of P. falciparum-invaded human RBCs. The in-plane shear modulus was calculated from the in-plane membrane displacement. Anatomic pathologists used changes in cellular morphology and tissue architecture to diagnose disease for a long time, as changes in morphology represent changes in cell state and function. For example, plasma membrane blebs can indicate dynamic cytoskeleton-regulated cell protrusions in apoptosis, cytokinesis, and cell movement^395^. Accordingly, diagnostic applications of holography focus on cell state to provide a diagnostic tool with early attempts using features from phase and RI to screen for cancerous tissue^396^. Besides, phase-measured viscoelasticity can differentiate between epithelial and mesenchymal states^397^. A state transition that is a cardinal feature of cancer cell metastasis, and phase correlation imaging discriminated between quiescent and senescent cells with potential implications for drug resistance and tumor reemergence^398^.

### Quantitative analysis tool for manufacturing

**Fig. 15:**
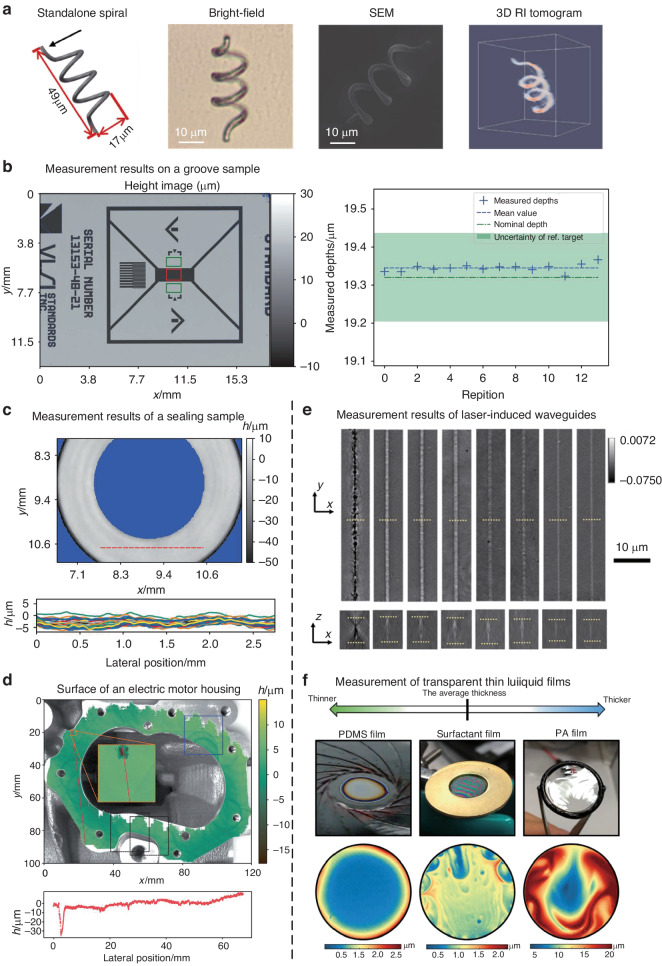
Holographic quantitative analysis tool of manufacturing. The complex-amplitudes of reflection waves reveal the profiles and topography of precision manufacturing. **a** Imaging results of a standalone spiral with bright-field image, SEM image, and 3D RI tomogram^404^ **b** Measurement results on a groove sample measured height values compared to the reference value from calibration data and uncertainty of reference measurement as given by the manufacturer of the sample^400^. **c** Measurement results of a sealing sample. **d** The measurement results of 30 consecutive measurements of the sealing surface of an electric motor housing. **e** The 3D RI distribution of the laser-induced waveguides in borosilicate glass^405^. **f** The measurement of weak absorption thin liquid films with different thicknesses and surface morphology^410^

Quality demands on mechanical and electrical components are increasing steadily, especially for mass-produced parts^170^, for example, in the automotive industry, medical technology, and chip manufacturing and assembly in the semiconductor industry. Tighter tolerances require very precise measuring methods. The demand for full-field inspection of functional surfaces is increasing. Reflective holographic imaging provides possibilities for the surface quantitative imaging of high-precision elements and devices. With increasing computing power and the availability of fast, high-resolution cameras, as well as the use of graphics processing units (GPU), the requirements for accuracy and manufacturing speed of today’s manufacturing cycles have been met. DH has become increasingly relevant for industrial applications^399,400^. In micro- and nano-device manufacturing, a few methods have been used to characterize structures by offering different resolving capabilities, including scanning electron microscopy (SEM)^401^, atomic force microscopy^402^, focused ion beam sectioning^403^, et al. However, these methods have various limitations, e.g., low measurement throughput, high sample invasiveness, sample contact, and small FOV. Most of the methods cannot obtain the internal features, but only the surface shapes. For example, SEM is frequently used for mapping surface shapes with a high resolution, but the sample often needs to be metal-coated to increase conductivity. Holography provides the ability of label-free representation of a specimen by measuring the scattered fields. Figure 15a shows a standalone spiral with a dimension of 49 × 17 × 17 μm^3^ and a period of 11.7 µm. The spiral was then imaged using bright-field microscopy and SEM, respectively. The whole spiral structure can be observed from the 3D RI by using ODT^404^. The structures revealed by ODT are consistent with the design, indicating the fabrication quality is high. Compared with bright-field microscopy and SEM, the entire 3D structure of the spiral can be revealed. It is very important to yield equal data quality throughout the FOV in high-precision 3D measurements. Figure 15b shows the test target of a quartz substrate with a chromium cover. The grooves of the test target had a depth of 19.320 µm. The uncertainty of the groove depths was given by the manufacturer as +/−0.116 µm^400^. The groove depth of the target was measured 14 times. They were calculated as the difference between the mean values measured in the two regions. One is the top of the groove target with green rectangles, and the other is inside the groove with a red rectangle. The mean value of the measured groove depths was 19.345 µm, and the standard deviation of all measured groove depths was 0.010 µm. The measurement of a sealing sample is shown in Fig. 15c. The gray area represents the points that were assigned to the central surface of the sample. The sample was measured 25 times. Between each measurement, the sample was picked up by the handling device, arbitrarily slightly rotated, and returned to the measurement position. The average value of this deviation was 8.1 µm and the standard deviation of the 25 measurements was 0.45 µm. The parameter changes and the performances in the 3D appearance of the sample manufacturing can be performed by holographic tomography. Figure 15d shows the 30 consecutive measurements of the sealing surface of an electric motor housing. A total FOV of 120 × 100 mm^2^ with sub-micron precision and lateral sampling of 7 µm was achieved. The general flatness of the sealing surface was only a few micrometers, but a flaw in the material extends by approximately 30 µm. Figure 15e shows the 3D RI of the laser-induced waveguides in borosilicate glass^405^. The fabrication process uses the transverse writing scheme in which the glass chip is moved transversely to the femtosecond laser beam on a 3D shifting stage. A series of power densities were employed to fabricate different optical waveguides. When the femtosecond laser power density was 4.19 × 10^6 ^mW/cm^2^, damage occurred in the glass samples, decreasing RI in the irradiation area. This localized reduction in RI can be further used to fabricate depressed cladding waveguides^406–409^. The term “thin film” is ubiquitous in our daily lives, and includes a broad range of materials, from solid to liquid films. The holographic technology has been demonstrated for the well adaptability in film measurements^410^. Figure 15f shows liquid films prepared using three different solutions: polydimethylsiloxane^411^, surfactants^412^, and polyacrylamide^413^. The thickness and morphology of different films can be revealed. It has shown excellent measurement capabilities for different materials, which are not only limited to physical properties, but also can successfully provide measurements for most thin-film materials. The above cases show enormous potential for holography. When applying these technologies to new fields of application, the major challenge is to further reduce the sensitivity of the interferometric technique to external influences such as relative movements between the sensor head and test specimen. Another bottleneck for wide-ranging industrial applications is the extensively frequency-stabilized laser sources. It is still necessary to find and characterize an appropriate laser source^414,415^.

## Multi-modal imaging

**Fig. 16:**
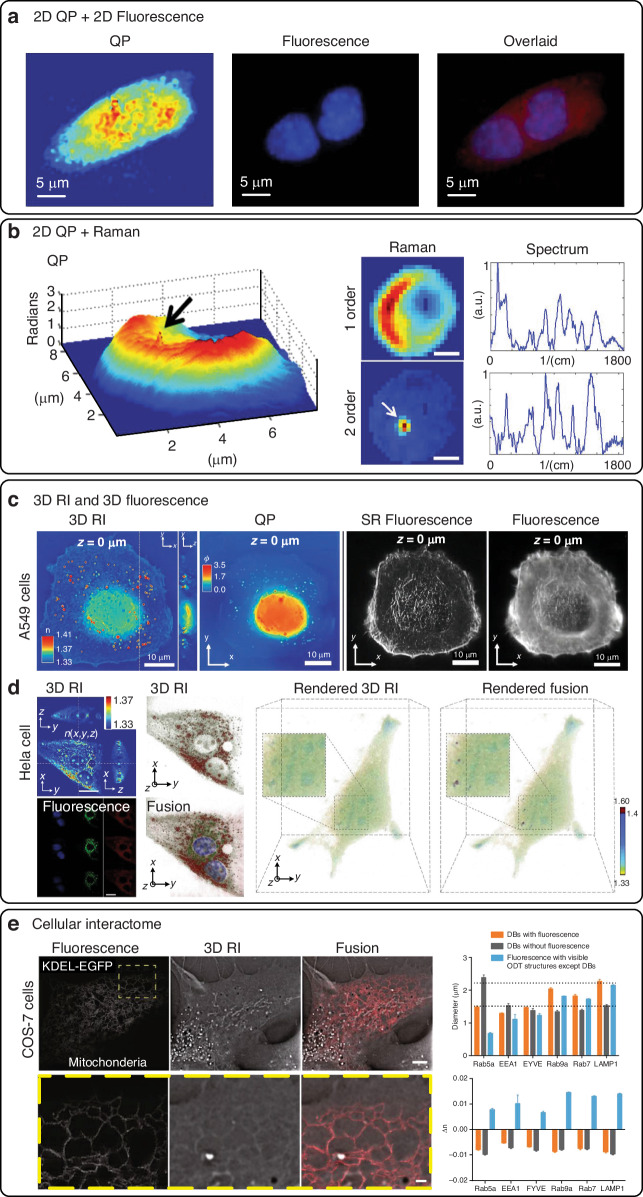
Coupling phase and RI with additional imaging modalities for multi-modal imaging. The key advantage of phase in cells is contributed from all components on cell mass, but they are not specific for any individual component. Other imaging modalities are combined with holography which help to learn more about cell structure and behavior for individual component. **a** Combining the quantitative phase of kidney cells with fluorescence detection enables the identification and quantification of dry mass changes^416^. **b** Combining the quantitative phase of red blood cells with fluorescence detection for monitoring cell development and cell-drug interactions^425^. **c** Enhanced fast image acquisition of 3D fluorescence and RI measurements of A549 cells from diffraction tomography^430^. **d** Combining 3D fluorescence with 3D RI measurements of HeLa cell^349,432^. **e** Super-resolution fluorescence-assisted RI diffraction tomography reveals the 3D landscape of the cellular organelle interactome^433^

A key advantage of phase and tomogram is that they are label-free and capture data on all components from cell mass. However, a related limitation is that phase reconstruction is not specific to any individual component of the cell. Several approaches and studies have combined phase or RI with other imaging modalities to learn more about cell structure and behavior. Early combinations of fluorescence detection methods with label-free phase approaches to interrogate RBCs measured physical and optical thickness, resolved substructures within cells, and identified and characterized the mass distribution of subcellular components^416–419^. As shown in Fig. 16a, the composite image using the quantitative phase and the fluorescence of a kidney cell becomes apparent in the process of mitosis. These initial approaches demonstrated phase identification and measurement of different subcellular components within a cell that were manipulated to fluoresce. Fluorescence combined with phase has also been used to segregate different populations of cells in a mixed culture experiment^386^, to track the behavior of rare subpopulations of primary human cells ex vivo^420^, or to determine different cell states concurrently with mass accumulation and mass density measurements from niche cell populations^397^. Dual fluorescence and phase combinations have also enabled biomechanical interrogations of cell responses to optical tweezers and dual traction force and growth measurements^421,422^. Another multimodal approach of interest is the combination of phase with molecular vibrational spectroscopy to measure chemical composition^423^. Molecular vibrational spectroscopy techniques generate vibrational spectra of molecules from their linear absorption and inelastic light scattering^424^. These vibrational spectra are dependent on the chemical structure and environmental interactions of the molecules. Raman spectroscopy is a type of vibrational spectroscopy, which relies upon the inelastic scattering of photons to determine the vibrational modes of molecules, allowing to confirm the abnormal shape and RI from the infected cells. Raman mapping was performed on a selected RBC which is a potential indicator for malaria infection^425^. Principal component analysis is applied for the case of healthy RBC, as shown in Fig. 16b. The first principal component closely resembles the hemoglobin spectrum. The second principal component with the corresponding spectrum shows a characteristic hemozoin peak at 1374 cm^−1^, which is clearly shown and confirms that hemozoin is localized in the region indicated by the arrow. Combined Raman and phase approaches have also examined dry mass, mass density, and protein and lipid composition under ultraviolet radiation^426^, and with the help of machine learning classified normal and cancerous tissues^427^.

In general, a combination of 3D RI tomogram and 3D fluorescence techniques can differentiate subcellular components while rendering a map of cell RI and identifying the RI of subcellular regions^428,429^. The holographic tomography module can resolve the mitochondria, lipid droplets, the nuclear membrane, chromosomes, the tubular endoplasmic reticulum, and lysosomes. Moving toward the acquisition of functional data from 3D structure, combinations of RI tomography with fluorescence sub-diffraction microscopy enable concurrent studies of cell biophysical properties and biochemical functions^418,430^, as shown in Fig. 16c. Further advances include high-speed correlative 3D RI and 3D fluorescence techniques, which have evolved to enable 200 Hz imaging of four-dimensional (4D, including 3D space and time) maps of cell structures^431^. By using 3D fluorescence microscopy. The nucleus, mitochondria, and actin are labeled with Hoechst 33432, green fluorescence proteins, and mCherry-C1, respectively, and they were excited with three sources with different spectra (center wavelengths are 385 nm, 470 nm, and 565 nm), respectively. As shown in Fig. 16d, the 3D RI distribution of cells clearly shows both the overall cellular morphology and the subcellular structures including the nucleus, nucleoli, and intracellular vesicles. The three-channel deconvoluted fluorescence images provide high molecular specific images, presenting nuclei, tubulin, and actin inside the cells separately. The multimodal method can also combine the advantages of ODT and 3D structured illumination microscopy by providing label-free 3D RI distribution with superior spatiotemporal resolution and molecular specificity^432^. The emergence of super-resolution fluorescence microscopy has rejuvenated the search for new cellular substructures. Combining 2D fluorescence Hessian structured illumination microscopy, novel subcellular structures named dark-vacuole bodies can be observed from the holographic tomography for a prolonged period^433^, as shown in Fig. 16e. The majority of which originated from densely populated perinuclear regions, and intensively interacted with organelles such as the mitochondria and the nuclear membrane before ultimately collapsing into the plasma membrane. As a label-free method, DH can be added to other microscopic imaging modalities beyond fluorescence and vibrational imaging methods. A combination of defocused phase and polarization microscopy can measure volumetric phase, retardance, and orientation, which is useful for studying structures in cells and tissue slices^434^. There is a broad potential future for multimodality work in biological and potential clinical applications of holography. With the addition of machine learning, more advances are possible due to the vast amount of morphological and molecular data collected by dual fluorescence and phase combination modalities, thereby enabling more complex analysis.

## Summary and outlook

**Fig. 17:**
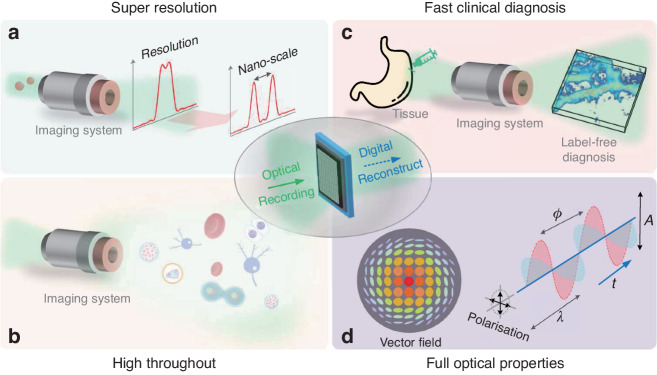
Future perspective about theoretical and application issues. **a** Toward super-resolution. An imaging system pushes the resolution toward the sub-diffraction limit by combining holography, microscopy, light scattering techniques, and vector diffraction theory. **b** Toward high throughout. bioimaging, It is expected to observe more dynamic events per minute. which is as critical as having a large FOV and a high resolution. **c** Toward fast clinical diagnosis. Using the advantage of label-free, DH can be further expanded in the classification and diagnosis of key cellular behaviors such as programmed cell death pathways, differentiation, cell cycle progression, and immunological responses. **d** Toward quantitative characterization measurement of full optical properties, where *t* is the time. Vector light fields describe the anisotropic structure sample. It brings our attention to a novel anisotropic feature of biological cells, micro- and nano-functional devices

The last two decades have seen great leaps in both the capabilities and applications of DH. The rapid recent development is due to impressive advances in image processing capabilities enabled by digitalization and increasing computational power. All the techniques and advances in the mathematical of DH is the around the transformation between complex-domain and real-domain. Then the data processing and analysis are developed based on the reconstructed wavefront described by the complex amplitude. Although holographic imaging has shown its great potential in the field of optical imaging and microscopy, as shown in Fig. 17, there are still several important theoretical and technical issues that deserve to be further studied:

Toward super-resolution: By combining holography, microscopy, and light scattering techniques, it can reach nanoscale sensitivity to morphology and dynamics, 2D, 3D, and 4D (that is, time-resolved tomography) non-destructive imaging of weak absorption structures. The quantitative signals are based on intrinsic contrast, which has emerged as a valuable method for investigating cells and tissues. Although super-resolution imaging of cells and subcellular organelles has been extensively shown using fluorescence tagging^435^, label-free super-resolution imaging remains a challenge. Synthetic-aperture approaches extended the maximum resolvable spatial frequency by a factor of two^216,250^, but it is still limited by the *NA* of the MO and the scalar diffraction theory. Super-resolution fluorescence-assisted holographic microscopy can reveal novel subcellular structures. An imaging system pushes the resolution toward the sub-diffraction limit while maintaining a large FOV, which serves as a vital tool to explore the connection between the molecular building blocks and overall tissue functionalities. It is expected that nonlinear interactions, such as pump–probe experiments, quantum physics, and quantitative imaging based on vector diffraction theory, may provide an opportunity for breaking the diffraction limit in label-free specimens^436^.

Toward high throughout: For bioimaging, the ability to observe fast dynamics is as critical as having a large FOV and a high resolution, particularly for in vivo or live-cell imaging applications. It relies on huge data processing capabilities and the compression optimization of algorithms. A direct expectation is to improve the temporal resolution without sacrificing the imaging SBP. It depends on the improvement of two aspects. One is to further improve the frame rate limited by the electronic data transfer rate from cameras to the host computers, which requires further development of hardware. Another aim is to reconstruct more information about the sample from a limited number of pixels as much as possible. Holographic multiplexing provides an effective idea for compressing more information into one hologram^173,230^, such as different DOF^183^, different spatial frequencies^212^, different FOV^194,230^, etc. Compressive sensing can also be used to compress more object information under some assumptions. It needs more optimization algorithms by jointing mathematical and physical models.

Toward fast clinical diagnosis: Current areas of holographic utility include studies of cell size and its regulation, cellular diagnostics, biomechanics, and biophysics. One key approach includes label-free classification of key cellular behaviors such as programmed cell death pathways, differentiation, cell cycle progression, and immunological responses. The benefit of imaging label-free specimens comes at the expense of losing specificity. Many researchers devoted extra efforts to integrating their instruments with the fluorescence modality, as described in the section on multi-modal imaging. The photobleaching and phototoxicity that come with it by using the fluorescence but the specificity is maintained. With the development of deep learning, the connections between the phase or RI with the specificity or physiological state of the subcellular structure become possible^347,348^. As techniques in cell profiling continue development, the increasing reports on molecularly distinct subpopulations of cells will be revealed. It provides a platform for assessing distinct phenotypes and behaviors within these heterogeneous populations.

Toward quantitative characterization measurement of full optical properties: Conventional DH and ODT consider the reconstruction of the mean phase in the sample. 2D and 3D optically anisotropic structures have been widely explored in the fields of cell biology, mesoscopic physics, material science, and industrial applications. Significant efforts have been devoted to the development of a variety of computational imaging modalities for structural and molecular interpretation of micro-scale transparent specimens. By modulating the polarization state of illumination, the optically anisotropic sample can be reconstructed such as polarization states^293,294^, birefringent scattering^295^, and dielectric tensors at optical frequency^437^. The intrinsic information about the scattered sample can be evaluated by measuring scattered vectorial light. It enables us to directly observe the 3D microstructures comprising anisotropic biomaterials such as DNA^438^, amyloid^439^, or collagen fibers in cancer tissues^440^. With the development of computational techniques, 3D anisotropic biological structures, and their orientational information are expected for quantitative characterization. Vector optics and vector diffraction theory may help to analyze the super-resolution and near-field anisotropic structures with label-free, serving detection and diagnosis of complex diseases, or micro- and nano-functional devices.

By reconstructing the complex-amplitude of the optical field, the interpretation of the phase signal has proven to deliver important novel parameters for studying physiological processes in living cells, such as transmembrane fluid flux, dry mass intracellular transport as well as tissue structure and density changes. The interpretation has also been proven for monitoring the wet etching process, and dynamic topographic changes at the nano-device, soft material, and industrial device. The technology is transferred from engineering to biomedical laboratories. Novel, high-impact, and some currently unexplored applications will surface. Commercialization efforts and application-driven academic collaborations across disciplines are key for these future developments.

The corresponding example code of different methods in this paper can be found in GitHub by live-updating mode^441^.
